# A conserved sequence signature is essential for robust plant miRNA biogenesis

**DOI:** 10.1093/nar/gkaa077

**Published:** 2020-02-06

**Authors:** Anushree Narjala, Ashwin Nair, Varsha Tirumalai, G Vivek Hari Sundar, Padubidri V Shivaprasad

**Affiliations:** 1 National Centre for Biological Sciences, Tata Institute of Fundamental Research, GKVK Campus, Bangalore 560065, India; 2 SASTRA University, Thirumalaisamudram, Thanjavur 613401, India

## Abstract

Micro (mi)RNAs are 20–22nt long non-coding RNA molecules involved in post-transcriptional silencing of targets having high base-pair complementarity. Plant miRNAs are processed from long Pol II-transcripts with specific stem-loop structures by Dicer-like (DCL) 1 protein. Although there were reports indicating how a specific region is selected for miRNA biogenesis, molecular details were unclear. Here, we show that the presence of specific GC-rich sequence signature within miRNA/miRNA* region is required for the precise miRNA biogenesis. The involvement of GC-rich signatures in precise processing and abundance of miRNAs was confirmed through detailed molecular and functional analysis. Consistent with the presence of the miRNA-specific GC signature, target RNAs of miRNAs also possess conserved complementary sequence signatures in their miRNA binding motifs. The selection of these GC signatures was dependent on an RNA binding protein partner of DCL1 named HYL1. Finally, we demonstrate a direct application of this discovery for enhancing the abundance and efficiency of artificial miRNAs that are popular in plant functional genomic studies.

## INTRODUCTION

MicroRNAs (miRNAs) are an evolutionarily conserved class of small RNAs (sRNAs) involved in the post-transcriptional regulation of long RNAs (1). In plants, most miRNAs induce classical ‘silencing’ by precise cleavage of target mRNAs leading to their degradation. A few miRNAs also induce translational repression of targets. However, in animals, most miRNAs induce translational repression and mRNA deadenylation that leads to their degradation (2). In addition to major differences at the level of regulation, biogenesis of plant miRNA is also different from its animal counterpart. Most miRNAs act as negative switches to regulate the expression of key genes such as transcription factors, thereby regulating development and stress responses.

Plant miRNAs mostly originate from intergenic miRNA (MIR) genes that exist as independent transcription units. Intronic miRNAs (known as mirtrons) and polycistronic or clustered miRNAs transcribed as a single transcript are less common in plants than animals with exceptions (3–6). Usually, evolutionarily conserved MIR genes also have conserved target gene families. Conserved miRNAs are usually expressed at high levels. There are at least 10 different miRNAs that are conserved across vascular plants whereas roughly 30 conserved miRNA families are conserved among flowering plants (7–9). On the other hand, almost all plants have less-conserved miRNAs that are likely to have less-conserved targets and generally are expressed at lower levels (10). A unique feature of these miRNAs is that they might share high homology with their target mRNAs beyond the targeting regions. Unlike conserved MIR genes, less-conserved MIR genes are present in fewer copy numbers, often one or two per genome (11).

Precise processing of Pol II transcribed primary miRNA (pri-miRNA) into miRNA duplex takes place in the nucleus where the components of processing complex form nuclear foci called dicing bodies (DB) (12). It has been proposed that pri-miRNA transcripts fold back due to internal sequence complementarity to form a hairpin structure called precursor miRNA (Pre-miRNA). The core complex in plant DBs consists of Dicer-Like1 (DCL1), an RNase III type enzyme; Hyponastic Leaves 1 (HYL1) or Double-stranded RNA Binding 1 (DRB1) and SE, a Zn-finger protein. DCL1 is the main enzyme that processes imperfectly complementary dsRNA in the nucleus. Other DCLs are involved in the processing of perfectly complementary dsRNA substrates, although DCL3 can also process such substrates *in vitro* (13). HYL1 and SE, in addition to DCL1, are required for precise and efficient processing of Pre-miRNAs (14,15). All these three proteins interact with each other (12).

DCL1 consists of helicase/PAZ/RNase III domains and two C-terminal dsRNA binding domains (15). HYL1 contains two dsRNA binding domain at the N-terminal followed by nuclear localization signal (16). HYL1 dimerizes through its second RNA binding domain which is required for its activity (17,18). HYL1 has been proposed to bind to the stem region and assist proper cleavage of pri-miRNA (18). Rarely, DCL1 can also partner with dsRNA binding protein 2 (DRB2), another dsRNA binding protein, to mediate miRNA biogenesis in *Arabidopsis* (19). SE consists of a core Zn-finger domain and terminal unstructured regions. SE can also bind to RNA, however, this property is not required to stimulate DCL1 activity *in vitro* (20).

In plants, the nature and composition of the core complex that processes pri-miRNA transcript and Pre-miRNAs appears identical. DCL1 dices the Pre-miRNA to release mature miRNA duplex of ∼21-nucleotide (nt) sRNAs. This dicing generates a 19-bp duplex with 2-nt 3′ overhangs. Occasionally, DCL1 complex measures length of miRNA depending on the presence of a bulge in the miRNA strand (21–23). Once the miRNA/miRNA* duplex is generated, it gets a protective 2′-*O*-methylation at the 3′ends by HUA enhancer 1 (HEN1) (24). miRNA duplex is believed to be exported to the cytoplasm by Hasty (HST), as mutants of HST had reduced the accumulation of most miRNAs in the cytoplasm (25), although recent reports suggest nuclear loading of miRNA duplex to AGO1, a key component of RNA-induced silencing complex (RISC) (26). AGO1 selects one strand over the other and mediates silencing of their targets.

Precise processing of miRNA from Pre-miRNA requires structural and sequence determinants that are not fully understood (27). Plant miRNA fold-backs are diverse in length and structure. miRNA precursors can be processed either base-to-loop or loop-to-base or bi-directionally, depending on the secondary structure. In longer stems, the processing is sequential producing one or more miRNA/miRNA* duplexes (28–33). Using a structure-function approach on selected miRNAs, three studies have shown that in base-to-loop processing, the first cut is made ∼15-nt upstream of a big bulge or ssRNA–dsRNA junction from the base (31–33). A sequence and structure conservation among miRNA precursors beyond the miRNA/miRNA* region has been reported from various species (34). Loop lengths in precursors that are processed loop-to-base were uniform, while loop length varied in base-to-loop processing precursors (34). The length of the loop itself might act as a determinant of miRNA biogenesis (35). Precursors with shorter loops of 20–50-nt are sources of more abundant miRNAs than those with longer loops. Asymmetric bulges and mismatches in miRNA/miRNA* duplex region in stem might lead to the formation of shorter 20-nt or longer 22-nt miRNAs (21,23). Pre-miRNAs are also processed to multiple miRNA-like sequences called sibling-miRs (sib-miRs) with unique sequences (36), or isomeric miRNAs (isomiRs), with slight variations at terminals compared to canonical miRNAs (37). These sib-miRs and isomiRs are usually low abundant and likely a result of inaccurate processing.

There are also a few sequence-specificity determinants of plant miRNAs. miRNAs with 5′-nt uridine (U) associate with AGO1 to form a functional RISC complex (38). This is the single-most important sequence determinant since more than 90% of all AGO1 bound miRNAs have 5′ U (38). Other sequence determinants are less obvious. It has been observed that dicot miRNAs have higher GC content than their precursor RNAs (39,40). In some precursors, tetra-nucleotide motifs like UCUC, AACA, GUGG, and ACGG are over-represented proximal to miRNA/miRNA* regions (41). Other bioinformatic analyses have indicated a bias for C at position 19 and A at position 10 in mature miRNAs (42). However, precise sequence and structural determinant rules applicable to all miRNAs are not known.

In this study, we show that plant miRNAs have unique GC content and a signature that is probably required for the selection of a precise region in the long precursor for DCL1-mediated processing. This GC signature is flexible but ensures multiple pockets of GC-rich regions across the miRNA/miRNA* regions. Our results also indicate that GC content and GC signature both play important roles in such a selection. The prominent signatures on precursors that determine efficient processing are G/C bias at positions 8–9, 18–19 and A/U bias at positions 5, 7, 10, 15. Less stringent signatures include G/C at positions 6, 21 and A/U at position 17, 20. Well-conserved mRNA targets of miRNAs have reciprocal complementary signatures, likely co-evolved for efficient targeting. We also show that the miRNA signature we identified in this study has the potential to enhance accumulation and activity of artificial (A)miRs that are useful tools in plant functional genomics.

## MATERIALS AND METHODS

### GC content analysis

Precursor miRNA and mature miRNA sequences were obtained from miRBase (version 21). miRNAs were separated into conserved and less-conserved miRNAs based on miRNA families. If a family is present in at least one member of both dicot and monocot plants then they are considered to be conserved miRNAs and if the family is specific to either monocot or dicot or species-specific, they are considered to be less conserved miRNAs. The GC content was calculated as the sum of the number of G and the number of C in a sequence, divided by the length of the sequence. Custom python scripts were used to calculate GC content and plots were plotted using R and/or Python. To analyze the GC content along the miRNA precursors, only those precursors were selected where both mature 5p and 3p sequences are annotated in miRBase, v21. For position-specific nucleotide analysis, only miRNAs of 21-nt or siRNAs of identical length were taken from the publicly available sRNA datasets (GSE28755) (7). The relative frequency of each nucleotide at position ‘x’ was calculated as the frequency of each nucleotide at position ‘x’ divided by the total number of sequences. This relative frequency was represented either as a fraction or as percentage numbers. The dinucleotide AU and GC relative frequency were also calculated in the same way. For abundant siRNA analysis, we used control datasets from Arabidopsis sRNA-seq dataset (GSE29802). sRNAs that are abundant (>10RPM) and aligned to the genome with zero mismatches from each size class was taken. From this list, the annotated miRNA sequences were removed and position-specific relative frequency of each nucleotide was calculated as mentioned previously and plotted using custom python scripts.

### Target GC analysis

Known targets of Arabidopsis miRNAs were curated from the literature and their sequences were obtained from the TAIR database. This set of target sequences and the miRNA sequences were used to determine the target position through psRNATarget software (43). Based on the obtained target site, a sequence of certain length before and after the target site on the mRNA was extracted. Distinct sequences of uniform length were taken and the relative frequency of nucleotides and the GC content along the length of sequences were calculated as a sliding window of 21-nt with a step of 1-nt. The position-specific GC content was normalized by dividing them by position-specific GC of random 21-nt sequences from cDNA sequences.

### Databases and online tools used

miRBase, version 21 (http://www.mirbase.org/) was used as the default miRNA database in this study. Primary miRNA sequences of *A. thaliana* were obtained from mirEX database (44). The non-coding RNA sequences were downloaded from the PNRD (Plant Non-coding RNA database) (45) for *Arabidopsis thaliana* and *Oryza sativa* species. The cDNA sequences of *A. thaliana* and *O. sativa* were downloaded from TAIR database (https://www.arabidopsis.org/index.jsp) and RAP-DB (http://rapdb.dna.affrc.go.jp/) (46) respectively. miRNA target regions on mRNA were predicted using either psRNATarget (47) (http://plantgrn.noble.org/psRNATarget/) or Tapir (http://bioinformatics.psb.ugent.be/webtools/tapir/). Tapir tool also used to predict mRNA targeting score and MFE ratios for miRNAs or amiRs. WMD3 (http://wmd3.weigelworld.org/) was used to design artificial miRNAs.

### *In vivo* assay for the GC preference by miRNA biogenesis machinery

An artificial precursor was designed with four stem-loops with identical sequence (modified miR156a of Arabidopsis), differing in only miRNA/miRNA* sequence. Each predicted mature miRNA has varying GC content and signature. This construct was synthesized and cloned into base vector pMK-RQ by GeneArt, Thermo Fisher, which was then subcloned into binary vector pBIN19, under 35S promoter. This construct was transiently expressed in *Nicotiana tabacum* leaves. Tissues were collected after 2 to 5 days post infiltration and total RNA was extracted using Trizol (Invitrogen) method. Equal amounts of RNA from both vector and artificial precursor infiltrated samples were resolved on 15% denaturing urea gel and transferred to the blotting membrane. Each mature sequence was probed using labelled complementary oligonucleotides (Supplementary Table S2). Secondary structures of the miRNA precursors were predicted using Mfold (48).

### Transient over-expression

Leaves of 3–4 weeks old *N. tabacum* cv. Wisconsin 38 or *N. benthamiana* plants were infiltrated with *Agrobacterium* strain LBA4404 with pSB1 (pSB1 harbours extra copies of *vir* genes for better efficiency, Stachel and Nester 1986) harbouring constructs to express genes of interest. The cultures of *Agrobacterium* were adjusted to OD_600_ = 0.7 using 10 mM MgCl_2_ (pH 5.6). Before infiltration, 60 μM of acetosyringone was added to the bacterial culture and incubated for 1 hour. Infiltration was carried out at the greenhouse where the temperature was maintained at 20°C (night) to 28°C (daytime). Infiltrated tissues were collected after 3 days for RNA analysis.

### Total RNA extraction, library preparation and sRNA-seq analysis

Total RNA was extracted from *N. tabacum* leaves expressing artificial precursor, collected at four different time-points after infiltration, using Trizol (Invitrogen) method. The concentration of RNA and purity of the samples was estimated using Nanodrop Spectrophotometer (Thermo Fisher Scientific) and Qubit Fluorometer (Thermo Fisher Scientific). sRNA sequencing libraries from 4dpi samples were prepared with TruSeq Small RNA Sample Preparation Guide (Illumina, San Diego, CA, USA) at Genotypic Technology Pvt Ltd, Bangalore, India, and sequenced on Illumina NextSeq500 platform. The sRNA-seq reads were processed for adapter removal and filtered for a length range of 16-nt to 35-nt using The UEA small RNA Workbench Version-3.2 (49). Processed reads were aligned to the artificial precursor using the PatMaN tool (50) allowing no mismatches and gaps.

### RNA blot analysis

The RNA blots were performed as described previously (51,52). Briefly, about 10- or 15-μg of total RNA extracted as mentioned earlier were dried and re-suspended in 8 μl loading buffer (0.10% bromophenol blue, 0.10% xylene cyanol in 100% deionized formamide), heated at 95°C for 1 min and loaded on to a 15% denaturing polyacrylamide gel (19:1 ratio of acrylamide to bisacrylamide and 8M urea). The gel was run at 80 V for ∼3 h and then transferred to a Hybond N^+^ membrane (GE Healthcare) by electro-blotting (Bio-Rad) at 10 V overnight at 4°C. Transferred RNAs were crosslinked using a UV crosslinker. The RNA hybridization was performed for 12 h using UltraHyb-oligo buffer (Ambion) containing appropriate radio-labelled short DNA oligo probes (Supplementary Table S4), end-labelled with ^32^P-ATP (BRIT, India) by T4 polynucleotide kinase (NEB) and purified through MicroSpin G-25 columns (GE Healthcare). The blot was washed twice with 2× SSC, 0.5% SDS for 30 min at 35°C. The signal was detected after exposure on a phosphorimager screen using a Molecular Imager (GE Healthcare). For repeated hybridization, the membrane was stripped and re-probed. Probes used in the analysis are listed in Supplementary Table S2.

### Small RNA analysis

Processed data of AGO1 IP sRNA (GSE10036) (38) and *hyl1-2* sRNA (GSE29802) (53) were downloaded and aligned to the *A. thaliana* genome using bowtie. Annotated miRNAs were identified using miRProf tool of UEA workbench.

### Sequence and structural analysis of dsRBD

HYL1 and other DRB sequences were obtained from the NCBI and Phytozome databases and aligned using the software clustalX. Crystal structure of dsRBD1 of HYL1 (PDB ID: 3ADG) was superimposed on frog dsRBD complexed with dsRNA (PDB ID: 1DI2), to identify the interacting residues.

### Construction of HYL1 knockdown constructs

For the antisense construct, 390 bp fragment from the 5′ end of *N. tabacum* HYL1 coding sequence was amplified (primers given in Supplemental Table 4) and cloned in reverse orientation into *Bam*HI and *Sac*I sites in pBIN19 background. The WMD3 tool was used to design artificial miRNAs against both the copies of HYL1 from *N. tabacum*. The selected amiR was then cloned into pMK-RQ vector by GeneArt, Invitrogen and was later subcloned into pBIN19 binary vector. These binary vectors were mobilized into *A. tumefaciens* LBA4404 (pSB1, with extra copies of *vir* genes) and used for infiltrations.

### Protein purification

Total RNA from A. thaliana Col-0 was isolated and converted into cDNA using Superscript III RT (Invitrogen). *At*HYL1 cDNA was amplified using Phusion High Fidelity Taq polymerase (Thermo Scientific) using appropriate primers that incorporate a C-terminal 6× His tag. The gel-purified PCR product was cloned into a pGEX-6P1 vector (GE Healthcare) that encodes an N-terminal GST (Glutathione S transferase) tag into *Bam*HI and *Xho*I sites. GST tagged *At*HYL1 (GST-*At*HYL1-6 × His) was transformed into Rosetta gami (DE3, Merck Millipore) cells. The culture was grown at 37°C until OD reached 0.7 followed by induction with 1 mM IPTG and incubation at 37°C for 5 h. The cells were lysed in lysis buffer (50 mM Tris–Cl, pH 8, 500mM NaCl, 5% glycerol, 5 mM DTT and protease inhibitor cocktail (Roche)) using an ultrasonicator for 15 cycles with 20 s ON/OFF each. The lysate was centrifuged at 16 000 rpm for 60 min and filtered through 0.45 μm cellulose acetate filters. About 5 ml of Protino GST/4B beads (Machery-Nagel) was equilibrated using equilibration buffer (50 mM Tris–Cl, pH 8, 250 mM NaCl, 5% glycerol, 5 mM DTT). The filtered lysate was slowly passed through the GST beads and the column was washed with equilibration buffer. Final elution was performed with 15 mM reduced glutathione. This GST purified protein was buffer exchanged to remove Glutathione. For further purification, GST tagged protein was bound to Ni-NTA (Qiagen) beads, washed thoroughly with high salt washes (1–2 M NaCl) and eluted with stepwise gradient of 100, 150, 200 and 250 mM Imidazole at pH 8. Elutes were further loaded onto a 15% denaturing reducing PAGE gel, fractions showing pure HYL1-GST was concentrated and used for assays.

For purification of double RNA binding domain of HYL1 (dsRBD12), the construct, dsRBD12 cloned with N-terminal 6× His tag in pET151/D-TOPO was transformed into Rosetta gami (DE3). The culture was grown and lysed in the same way as mentioned above for GST-*At*HYL1-6× His. About 20 mM Imidazole was added to the clarified lysate and was applied to pre-equilibrated Ni-NTA beads. The beads were then thoroughly washed with high salt wash buffer (50 mM Tris–Cl, pH 8, 1 M NaCl, 5% glycerol and 20 mM Imidazole at pH 8.0). Protein was eluted with a stepwise gradient of 100, 150, 200 and 250 mM Imidazole pH 8.

### Electrophoretic mobility shift assay

EMSA was done as described previously (54) with some modifications. Briefly, 250 ng of annealed RNA was labelled with ATP γ-32P (BRIT, India) in 20 ul reaction with T4 polynucleotide kinase (NEB). Before the reaction, labelled products were diluted equally to 50 CPS (counts per second). An equal amount of diluted RNA was used for each reaction. Soluble purified protein or peptide (Supplementary Table S3) was incubated with labelled oligos (Supplementary Table S4) in binding buffer (Tris pH 8, 83 mM, NaCl 100 mM, KCl 66 mM, MgCl_2_ 5 mM, DTT 5 mM) at 25°C for 30 min, after which the reaction was stopped by adding loading dye containing EDTA. This was further loaded onto a 4% TBE gel that was electrophoresed at 6 V/cm under cold conditions. After electrophoresis, the gel was exposed to a Phosphor screen (GE) and imaged using Typhoon trio scanner (GE healthcare).

### Artificial miRNAs

The WMD3 designer tool was used to get a list of amiR candidates for *Vv*MYBA7 and GFP targets. From this list, presumably best candidates (encoded by green colour) were checked for variations in GC content and signature. The candidates were chosen based on their GC content variations. The star sequences of each amiR candidate were also designed using WMD3 Oligo tool. The amiR and star sequences were inserted into the miR319 precursor replacing the original miR319/* sequence, and *Ba*mHI-*Sac*I sites were inserted at the ends of pri-miRNA. These constructs were synthesized and cloned into base vector pMK-RQ by GeneArt, Invitrogen, which was then subcloned into binary vector pBIN19, under 35S promoter. The amiRs were co-infiltrated with their targets.

### Anthocyanin estimation

Leaf sections of 6 days post infiltration were used for anthocyanin estimation. The method was performed as described previously (55). About 5 volumes of extraction buffer (45% methanol and 5% acetic acid) were added to the plant tissues and mixed thoroughly. Centrifugation was performed twice at 12 000 g for 5 min at RT. The absorbance of the supernatant was measured at 530 and 657 nm and anthocyanin content were calculated by using the formula (Abs530/g F.W.) by [Abs530 – (0.25 × Abs657)] × 5.

## RESULTS

### A conserved GC enrichment among plant miRNAs

We hypothesized that a structural and/or a sequence determinant might be contributing to the selection of a specific region in the Pre-miRNA for further processing. To identify a conserved sequence determinant across miRNA precursors including those from animals, we used all miRNA entries in miRBase (version 21) (56). In this analysis, we observed that plant miRNAs had considerably higher GC in their miRNA/miRNA* regions than their precursors (Supplementary Figure S1A). Higher GC in mature miRNAs was not observed among animal miRNAs except in some invertebrates. Due to the high genome GC content of monocots, especially *Poaceae* family members that have high genome GC (57), a GC-bias was not easily distinguishable. These observations matched a previous report from Ho *et al.* (40,58), performed with fewer miRNAs from selected species, instead of a robust global analysis.

**Figure 1.**
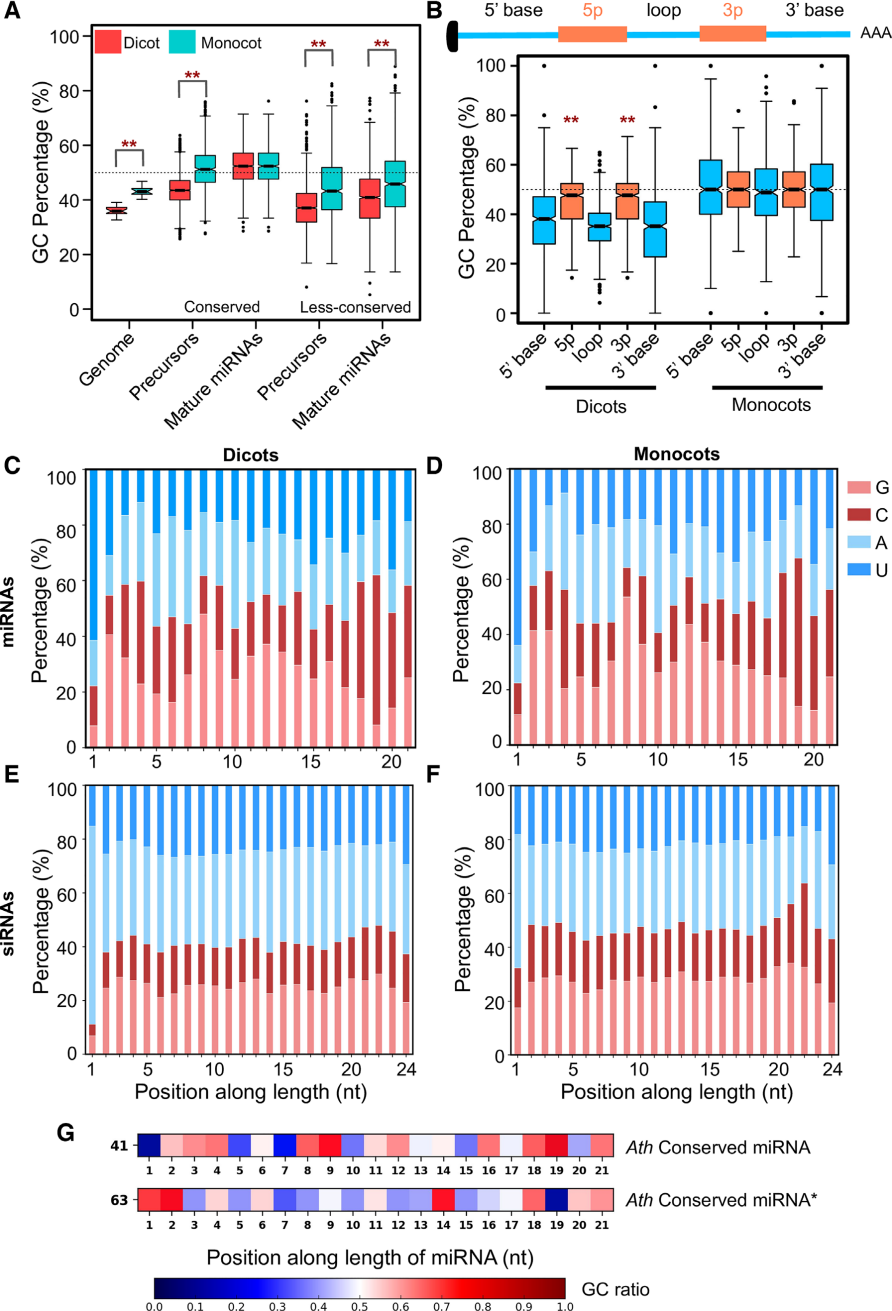
Plant mature miRNAs have unique GC content and signature. (**A**) Boxplots representing GC content of Pre- and mature miRNA regions of conserved and less-conserved miRNAs (dicot genome, 20; monocot genome, 14; dicot conserved precursor, 2335; miRNAs, 2722; monocot conserved precursor, 691; miRNA, 1005; dicot less-conserved precursor, 2471; miRNA, 2862; monocot less-conserved precursor, 925; miRNA, 1191). Boxplots display interquartile range (box), whiskers (extending 1.5 times the interquartile range), median (thick line), and outliers (dot). The notch around median represents a 95% confidence interval. Asterisk labels significant difference (**P < 0.001, Mann–Whitney *U* Test) between dicot and monocot GC contents. (**B**) GC content in different segments of Pre-miRNAs from monocots and dicots (dicots, 769; monocots, 557). The asterisks indicate significant difference (**P < 0.001, Mann–Whitney *U* Test) between mature miRNA GC and precursor GC of dicots. (**C–F**) Position-specific percentage nucleotide abundance, along the length of conserved unique dicot miRNAs (672), (C); conserved unique monocot miRNAs (263), (D); 24-nt sequences from dicots (546375), (E); 24-nt sequences from monocots (265546), (F). (**G**) Matrix showing flexible GC signature across miRNAs (5′ to 3′). Non-redundant conserved 21-nt miRNAs from *A. thaliana* (41), and miRNA stars of conserved miRNAs from *A. thaliana* (63) were used. The colour represents the GC ratio at each position.

We next selected two diverse plants, namely *A. thaliana* and *O. sativa*, for comparison of GC enrichment (Supplementary Figure S1B). Only tRNAs (GC at 55%) and miRNAs (52% GC) from *A. thaliana* had higher GC than average mRNA GC (42%). Similar GC-bias was also seen for *O. sativa*, albeit, due to high genome GC, mRNAs also had GC% of around 53%. Interestingly, GC-bias was limited to conserved and highly expressed miRNAs in both plants. GC% of less-conserved miRNAs that are low abundant and clade/family-specific, was closer to mRNAs, CDS or genome GC (Supplementary Figure S1B and Figure 1A). These observations imply an evolutionary selection for higher GC content for miRNAs similar to tRNAs.

We explored if higher GC is uniformly distributed across the pri-miRNAs, or limited to Pre-miRNA or miRNA/miRNA* regions. Surprisingly, higher GC was limited to miRNA/miRNA* duplex regions than stems and loops (Figure 1B and Supplementary Figure S1C). However in monocots, there was no significant increase or decrease in GC content of miRNA regions compared to the rest of the precursors, likely due to the high genome and mRNA GC of grass family described previously (Figure 1B, Supplementary Figure S1D–F). Less-conserved miRNAs across dicots and monocots had a tendency to have higher GC than stems and loop regions (Supplementary Figures 1D, E).

In order to verify a preferred GC content for mature miRNA/miRNA* regions, we used publicly available sRNA datasets to look for the abundance of mature miRNAs in comparison to isomiRs and sib-miRs. In miRNA families such as rice miR444, a less-conserved miRNA, at least three sib-miRs were generated, likely due to the presence of a long stem region (Supplementary Figure S2A and B). While multiple miRNAs were generated as sib-miRs having more than three sliding positions between them, the most abundant forms had a GC content of ∼52%. These results indicate that a specific GC content of ∼50% is preferred for all miRNAs that are abundantly expressed in plants.

### A miRNA-specific, flexible GC signature

A major criterion for miRNA nomenclature is its unique sequence. It is unlikely that miRNAs share sequence similarity between families in order to maintain a GC signature. In order to understand the basis for GC-bias in miRNAs, we used all conserved miRNA entries from dicots and monocots and examined position-specificity. Majority of miRNAs, as expected, had a U bias at the 5′ end. However, we also observed bias for G or C in few pockets such as positions 2–4, 8–9, as well as positions 18–19 and 21, compared to the adjacent sequences (Figures 1C and D, Supplementary Figures S3, S4A and B). Randomly sampled miRNA sequences also showed a bias, whereas the dinucleotide shuffled sequences did not show any preference, suggesting the observed bias in positions is not by random chance (Supplementary Figures S4C–F). In some positions especially among dicots, there was a clear AU-bias, such as in positions 5, 7, 15 and 17. The position-specific GC preferences were conserved among dicot and monocot miRNAs (Figures 1C and D). As expected, in redundant reads of conserved-miRNAs across dicots and monocots, these positions were enriched with G or C often more than 80% (Supplementary Figures S5A and B).

We then explored if specific signatures are common among other classes of abundant sRNAs, such as siRNAs. GC preference was not observed among abundant 21-nt reads other than miRNAs as well as reads of 22- and 24-nt (Supplementary Figures S6A-C), confirming that GC-bias was not associated with sRNA length. In such reads, there was a preference for A or U with a higher preference for G or C in the terminals, such as in positions 2, 3, 19 and 20. This preference for G or C at the terminals was also observed previously among viral siRNAs (59). The 24-nt siRNAs, specifically those derived from transposons, accumulate to higher levels than miRNAs in most plants. In both highly abundant reads of 24-nt and unique 24-nt reads, we did not observe any bias for any position, except the well-known 5′-nt bias for Adenine (A), due to their association with AGO4 (Figures 1E and F, Supplementary Figures 5C and D). Reads of 24-nt had a strong preference for A or U, since the sources of such 24-nt RNAs are AU-rich transposons and repeats (60). Correspondingly, transposon and transgene-derived siRNAs are unable to target GC-rich coding sequences as observed recently (61).

Similarly, there was an absence of bias for any position among 21-nt phased secondary siRNAs named tasiRNAs (Supplementary Figures S6D–F). The abundant tasiRNAs had a preference for U at the 5′ terminal end (Supplementary Figure S6F), indicating their association with AGO1. Since tasiRNAs also associate with AGO1, it is unlikely that the GC-bias we observed among miRNAs was due to AGO1-binding specificity. In agreement with this, sRNAs enriched in AGO1-Immunoprecipitation (IP) datasets did not show any GC-bias except for miRNAs (Supplementary Figure S7). In specific positions across miRNAs that are likely to be biased for G or C, the possibility of having G or C was not absolute, indicating that several miRNAs have A or U in those positions. We observed that specificity for G or C at such positions was less obvious and flexible with respect to its neighbouring base while maintaining the overall GC of the miRNA to ∼50%.

In support of the observations made above, conserved and abundant miRNAs from *Arabidopsis* matched the GC position-specificity, although there were minor variations in one or two positions in some miRNA variants (Figure 1G and Supplementary Figure S8A). As expected, the miRNA* sequences had complementary GC signature of miRNAs with a 2-nt shift (Figure 1G). In addition, this signature was independent of miRNA processing mechanisms (loop-to-base or base-to-loop), since miRNAs from these two groups showed almost identical expected GC signatures (Supplementary Figure S8B–D). In line with this, the observed signature was well-conserved across the green-plant lineage, especially among well-conserved miRNAs (Supplementary Figures S9 and S10). In all miRNAs, elevated GC and GC signature was observed only in miRNA/miRNA* regions, but not in the neighbouring 21-nt regions where there was a clear absence of any positional bias (Supplementary Figure S11). Together, these results indicate a specific preference for G or C in many positions of miRNAs so that an average GC content is maintained. Such a preference was not observed among other categories of abundant sRNAs.

### Target mRNAs harbour complementary GC signatures at miRNA-binding sites

**Figure 2.**
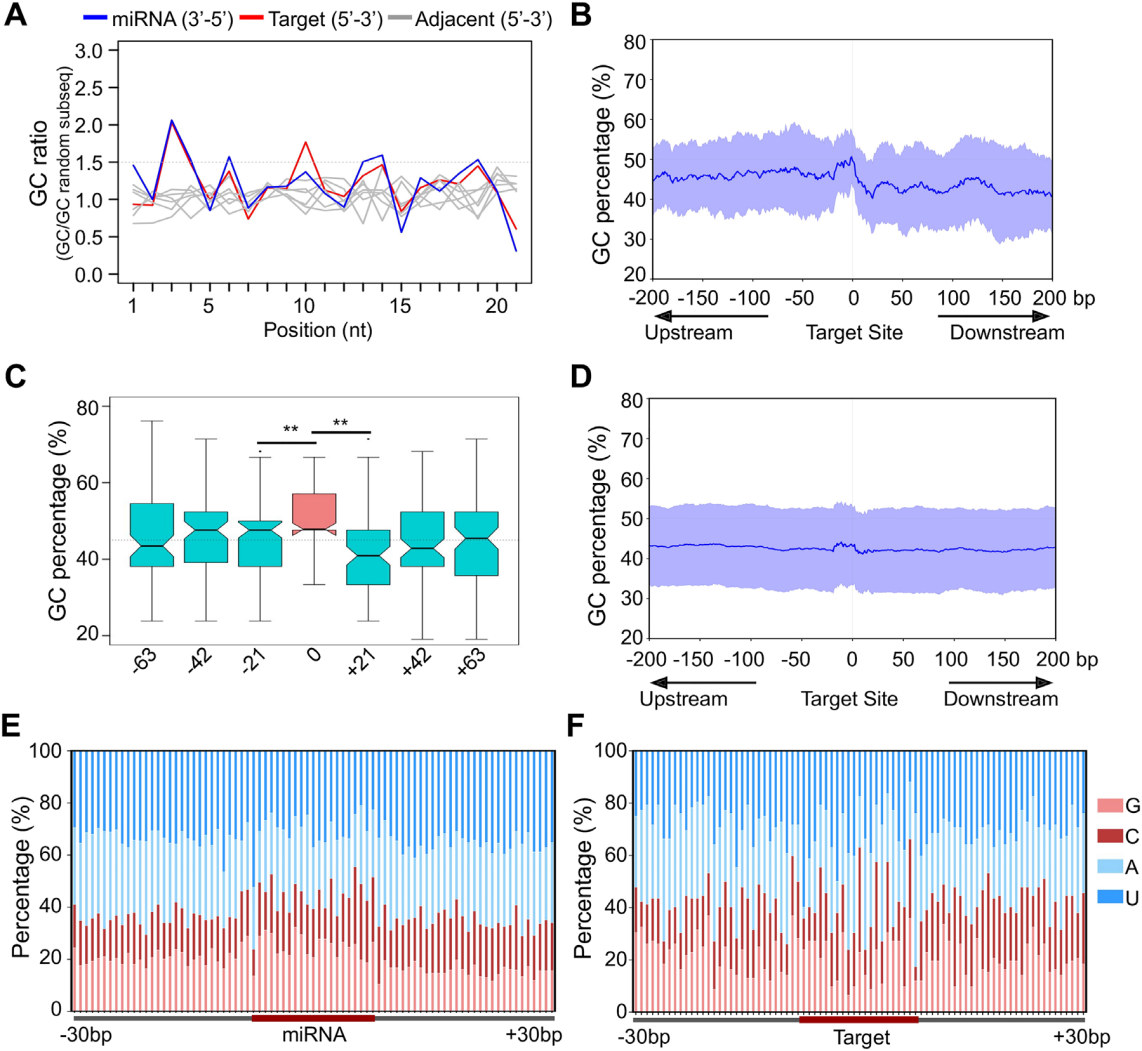
miRNA target sites in mRNAs complement miRNA GC signature. (**A**) Position-specific GC ratios of miRNA target regions/average cDNA GC content (85, non-redundant target sequence). Blue: miRNA, Red: target RNA, grey: an adjacent 21-nt window on the target RNA. (**B**) GC content across conserved miRNA target sequences (145) of *A. thaliana* that are experimentally verified. Mean and standard deviation of GC content around the target region was calculated as the sliding window of 21-nt represented as the blue line and light blue region, respectively. (**C**) GC percentages at three consecutive 21 nt windows before and after the target site (**P < 0.001, Mann–Whitney *U* Test). (**D**) GC content across miRNA target representative gene model sequences of *A. thaliana* that are predicted by the psRNA-Target tool (e-value cutoff of 4, 3379). (**E**) Position-specific GC signature of conserved miRNA across precursors in the 30-nt window (353 unique miRNAs from *A. thaliana*). (**F**) Position-specific GC signature in miRNA target regions (92 unique targets).

Plant miRNAs require a higher degree of complementarity with their target RNAs for efficient targeting. This is likely due to their co-evolution with their target RNAs to maintain efficient targeting to ensure proper development. It is hypothesized that plant miRNAs arose due to duplication of their target gene and subsequent incorporation of sequence variations through mutations (62). A conserved miRNA is likely to have complementarity only with the target region, whereas, less-conserved miRNAs have complementarity extending beyond miRNA target regions (63). We explored if the RNAs targeted by miRNAs have a GC signature to maintain complementarity with miRNAs. We analyzed sequences of 145 targets of conserved miRNAs derived from *A. thaliana* that are proven miRNA targets based on degradome data (64,65). In target regions of all these mRNAs and non-coding RNAs, a complementary GC-bias matching with the GC signature of miRNAs was observed (Figure 2A). The GC-bias in target RNAs were very specific in all mRNA positions matching miRNA signatures, except in position 12 of miRNA (or complementary position 10 of target) where target RNAs had high GC preference that is not complementary to miRNA signatures (Figure 2A). Significance of this non-complementarity region is not known, however, this observation offers rich possibilities for future research. Adjacent regions of target RNAs did not harbour sequence signatures, suggesting that mRNAs complemented miRNA signature only in the target regions.

We observed a uniform distribution of GC and AU across RNAs except in the RNA motifs where miRNAs targeted (Figures 2B and C). This trend was also seen when all predicted targets of conserved miRNAs of *Arabidopsis* or *O. sativa* were used in the analyses (Figure 2D and Supplementary Figure S12A). In general, both extended miRNA regions and extended miRNA target regions on mRNAs exhibited GC signature at a 21-nt window and this signature was complementary to each other (Figures 2E and F, Supplementary Figure S12B).

### Artificial RNA substrates with varied GC content are processed differentially

**Figure 3.**
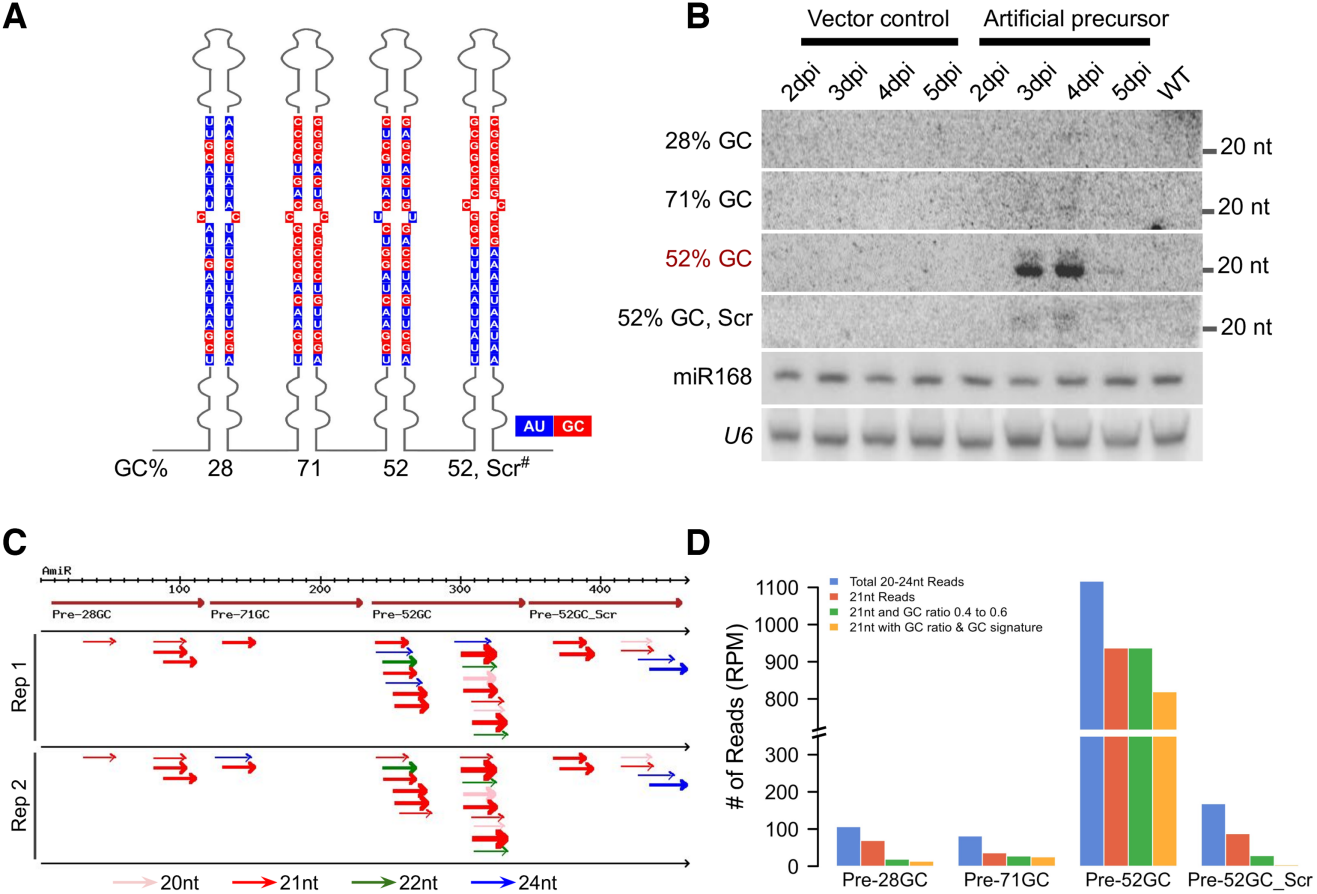
Efficient GC-content-specific processing of miRNA precursors *in vivo*. (**A**) 2D representation of artificial precursor with four stem-loops, each having unique mature sequences of varying GC content as indicated. The highlighted region represents miRNA/miRNA* region. AU marked in blue and GC in red. This construct was expressed under the CaMV 35S promoter. ‘Scr^#^’ - scrambled signature. (**B**) Northern analysis of miRNAs with different GC content in *N. tabacum* leaves. *U6* and miR168 served as loading controls. (**C**) Sequenced sRNAs from *N. tabacum* leaves expressing artificial precursor at 4 dpi, were mapped to the precursor. Four big brown arrows indicate the four stem-loops in tandem. Pink, red, green and blue represent 20, 21, 22 and 24-nt reads, respectively. The thickness of the arrow is indicative of the abundance. Only reads sequenced more than 5 RPM are considered. (**D**) Graph comparing the abundance of sRNAs mapped to the artificial precursor. Blue (all reads of 21–24-nt), red (21-nt), green (reads that have GC content between 40% and 60%), yellow (reads that have GC content between 40% and 60% and also GC signature) bars indicate abundance.

The presence of GC-bias indicates that plant DCL1 machinery might prefer such a signature. If DCL1 complex mediates such a preference, substrates with miRNA-like GC signatures are likely favoured for processing. In order to validate the presence of a conserved GC signature within miRNAs, we transiently expressed in *N. tabacum*, substrates having sequences of varied GC content by swapping sequences of a conserved miRNA. In order to minimize the bias for transcription, four stem-loop structures with identical sequences except in miRNA/miRNA* regions were driven by CaMV 35S promoter (Figure 3A, Supplementary Figure S14A). As expected, control tissues with vector-alone construct did not accumulate sRNAs at detectable levels in northern analysis. However, miRNAs of 52% GC accumulated at very high levels in a predominant 21-nt form (Figure 3B). A structure also with 52% GC but with GC biased only at one end of the mature miRNA region accumulated low levels of sRNAs including non-canonical sized ones. Also, miRNAs of 28% and 71% GC accumulated at negligible levels.

We generated sRNA datasets from the agroinfiltrated tissues (Figure 3C) to understand the nature of such a bias and to confirm the results of RNA blot analysis. When reads were mapped to the introduced construct, we found low abundant reads spanning all four stem-loop regions indicating that the precursor RNA was indeed expressed in *N. tabacum* (Supplementary Figures S13A–D). Confirming the observations in northern analysis, there was a massive accumulation of miRNAs of 52% GC (Figures 3C and D) in sRNA datasets. Most reads matching 52% GC stem were of 21-nt length and also nearly 60% of such reads had signatures of GC (Figure 3D, Supplementary Table S1). These observations confirm the presence of a GC signature in plant miRNAs that host DCL1 complex specifically process to generate abundant miRNAs.

### GC content and GC signature both play a role in miRNA processing

**Figure 4.**
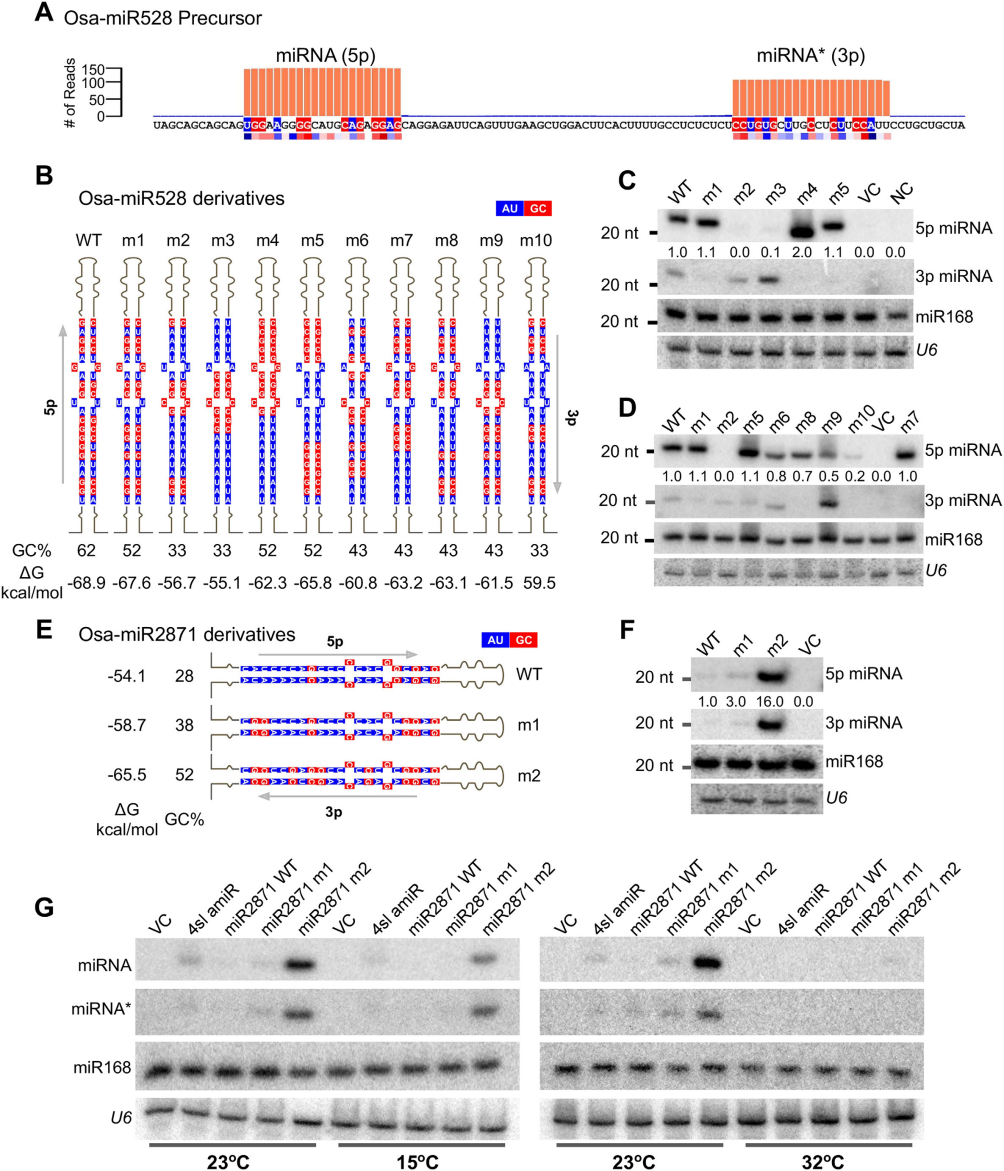
Both GC content and GC signature are required for proper processing *in vivo*. (**A**) Schematic representation of WT Osa-miR528 precursor showing the precise processing and abundance of miRNA and miRNA*. Abundance was taken from GSE111440. The red and blue coloured strip below the sequence is the observed GC signature from conserved miRNA/miRNA* sequences (Figure 1G). The residues matching the signature are coloured likewise. (**B**) Schematic representation of miR528 derivatives, highlighting mutated miRNA/miRNA* sequences. Percentage of GC in miRNA/miRNA* regions are indicated. Blue represents A/U and red represents G/C. (**C** and **D**) Northern blot to probe abundance of WT miR528 and its derivatives in infiltrated *N. tabacum* leaves. Equimolar probes against all miRNAs were mixed, labelled and probed. *U6* and miR168 served as loading controls. (**E**) Schematic representation of miR2871 derivatives, highlighting mutated miRNA/miRNA* sequences. Percentage of GC in miRNA/miRNA* regions are indicated. (**F**) Northern blot to probe abundance of WT miR2871 and its derivatives in infiltrated *N. tabacum* leaves. Equimolar probes against all miRNAs were mixed, labelled and probed. *U6* and miR168 served as loading controls. (**G**) Temperature sensitivity of amiR biogenesis. Northern blots to probe abundance of abundant amiR forms and their derivatives in *N. tabacum* infiltrated leaves at three different temperatures (15, 23 and 32°C).

In the above experiments, we could confirm the presence of a GC signature with approximately 50% GC content in plant miRNAs. However, it was not clear if GC content is more important than position-specific GC signature for processing and therefore the abundance of miRNAs. In order to understand the hierarchy of importance of these features, we introduced monocot-specific miR528 that has the expected GC signature (Figure 4A), along with its derivatives in *N. tabacum* where it is not naturally expressed (Figure 4B). miR528 is precisely processed in all plants where it is expressed including rice (Figure 4A). All derivatives of miR528 precursor had predicted secondary structures similar to miR528 (Supplementary Figure S14C). As expected, *N. tabacum* did not accumulate detectable levels of miR528 in control samples (Figure 4C). Mature miR528 that has slightly higher GC content at 62%, accumulated at slightly lower levels than miR528 mutated in 2 positions (m1) to bring down the GC% to 52% (Figure 4B). miR528-m1 version had A or U in positions 7 and 10, to match the optimum GC signature when compared to WT miR528. This indicated that both GC content and signature are important at least for miR528. Other 52% GC-containing miR528 versions without signatures, accumulated to the level of m1, but processed inaccurately to produce shorter sRNAs such as in m4 (Figure 4C). On the other hand versions with low GC and minimal GC signatures were barely detectable (m2 and m3, Figure 4C).

In order to further clarify the role of GC signature, we introduced additional constructs that have identical GC content, but with varying position preference for G or C (Figure 4B and D). In this analysis, it is clear that GC signatures at positions 8 and 9 as well as positions 18 and 19 are absolutely required for abundant processing of these miRNAs. For example, in construct m7 where at positions 8 and 9, as well as at positions 18 and 19 have G or C, but having AU rich sequences in the 5′ end still produced abundantly processed sRNAs, indicating that pockets of GC at these positions are more important than GC pockets at positions 2–4 (Figure 4C and D). In these constructs, GC content was maintained at 43%. In another derivative, m10 (where GC signature at positions 2–3, 8–9, 18–19 and 21 was kept intact while the overall GC reduced to 33%) accumulated at very low levels. Even when the GC content is similar, retaining GC signature increased their accumulation as seen in a comparison between m2 (33% GC, without a signature) and m10 (33% GC with signature).

To check if, among the low abundant miRNAs, the star sequences had accumulated disproportionately, we probed for miRNA* (3p) sequences. In mutants where miRNA was less as in m2, m3, and m9, the star sequences had accumulated at slightly higher levels but these were still very low abundant, indicating that strand selection was not the major reason for the higher abundance of sequences with GC signatures. miR528-m10, even though had low 5p abundance, did not accumulate 3p (Figure 4C and D). These results suggest that both GC content and GC signature play a role in miRNA biogenesis and their accumulation. With this, we conclude that the GCs at positions 8–9, 18–19 and AUs at positions 5, 7, 10 and 15 are the minimal signature that is required for efficient processing and GCs at positions 2–4, 6 and 21 are comparatively less important.

In order to verify the importance of GC signature, we introduced another less-conserved miRNA from rice (miR2871) that is generally expressed at lower levels but induced during various abiotic stresses (66). Interestingly, this miRNA has low GC (28%). We introduced WT miR2871 as well as its derivatives having increasing GC content when compared to WT, including a construct having optimal GC content and signature (m2; Figure 4E) into *N. tabacum*. In all these constructs the backbone was identical (Supplementary Figure S14B). As expected, miR2871 version m2 having GC% of 52 with signatures such as G or C in positions 2–4, 6, 8–9, 18–19 and A or U at positions 5, 7 and 10 produced abundantly processed miRNAs (Figure 4F). In construct m2, there was at least 10-fold higher accumulation of miRNAs when compared to WT miR2871. In m2, accumulation of miRNA* (3p) sequence was high and proportional to miRNA (5p) levels., As per our prediction, the 3p sequence starts with 5′ nucleotide G, and it is unlikely that AGO1 bound and selected this sequence. If GC signature was required for strand selection then we would have seen less abundant star (3p) sequence. These results indicate that GC signature helps with the biogenesis of abundant miRNA:miRNA* duplexes.

It is well known that sRNA biogenesis in general, and miRNA biogenesis and accumulation in particular, are temperature-dependent (67,68). To evaluate if the requirement of GC signature was also dependent on optimal temperatures, we performed infiltrations at three different temperatures. We introduced the above-mentioned constructs and their derivatives into plants grown at low (15°C), optimum (23°C) and high (32°C) temperatures. In all cases, miRNAs accumulated at low levels in 15 and 32°C when compared to optimal 23°C and the trend to select optimal GC remained the same across all tested temperatures, indicating that the effect of GC signature-based selection on miRNA biogenesis is temperature independent (Figure 4G).

### GC signature is associated with HYL1 binding preference

Several proteins act as accessories of DCL1 in plant miRNA biogenesis. Very few proteins specifically bind to Pre-miRNAs. It has been previously shown that specific residues in HYL1 dsRNA-binding domain 1 (dsRBD1) are essential for substrate binding (17,69). Previous reports suggest that the processivity of DCL1 is compromised in *hyl1* mutants, resulting in low-abundant sib-miRs that are diced randomly from the precursor (15,18). Analysis of WT and *hyl1* mutant-derived sRNA datasets indicated that processivity is indeed lost in mutant and that miRNAs accumulate at negligible levels (Supplementary Figures S15A and B). Among dsRBDs, HYL1 RBD has certain unique residues adjacent to nucleotide binding residues conserved among other homologue from various plants (Supplementary Figures S16A and B). These residues are conserved among HYL1 homologues from various plants (Supplementary Figure S16E). We hypothesized that HYL1 might be a candidate that can potentially select a signature across the precursor so that DCL1 can accurately generate a unique miRNA/miRNA* sequence.

**Figure 5.**
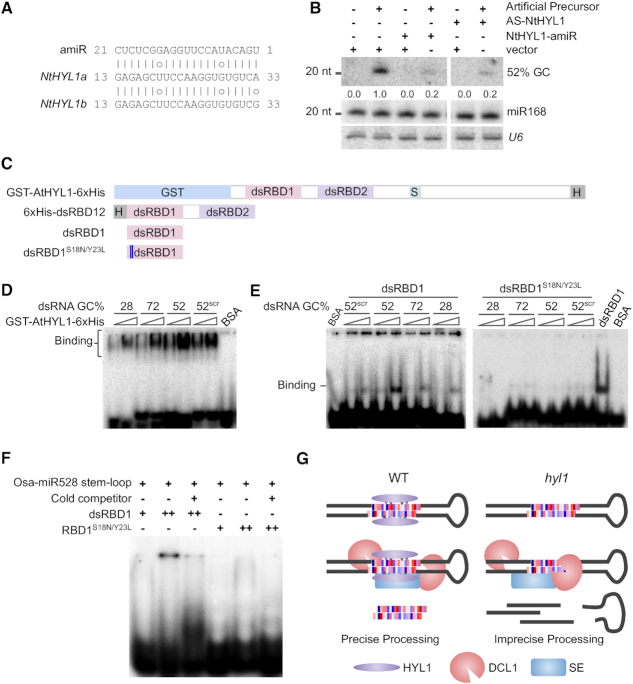
Specific mutations in dsRBD1 of HYL1 affect specificity of RNA binding. (**A**) Complementarity between *Nt*HYL1-amiR and it's target site on two homologs of *Nt*HYL1. (**B**) Northern blot probed for the abundance of 52% GC miRNA after co-infiltration of *Nt*HYL1-amiR or antisense (AS) - *Nt*HYL1, with an artificial precursor in *N. tabacum* leaves. *U6* and miR168 were served as loading controls. (**C**) 2D domain structure of *At*HYL1 and its derivatives used for biochemical assay. (**D** and **E**) EMSA with full-length *At*HYL1 (E), and dsRBD1 (F). Annealed 21-nt RNA duplex with varying GC (mature forms of artificial precursor with four stem-loops, Figure 3A) was used as substrates. The binding was tested with increasing concentrations of dsRBD1 WT and dsRBD1^S18N/Y23L^ peptides. BSA was used as negative control and 52% GC duplex was used as the substrate in the lanes where it is not mentioned in the figure. (**F**) EMSA with WT and mutated dsRBD1 peptide. Modified Osa-miR528 stem-loop used as substrate. (**G**) Proposed model for precise selection of miRNA by HYL1 and DCL1.

In order to understand the involvement of HYL1 in selective processing of precursors, we silenced *Nt*HYL1 with artificial miRNA (Figure 5A) as well as by using an antisense construct. In amiR targeted lines as well as antisense-silencing lines, levels of HYL1 mRNA was reduced, confirming targeting of endogenous HYL1. We infiltrated artificial precursor with 4 stem-loop structure (Figure 3A) into the leaves of these plants and analyzed for differential processing of miRNAs with varying GC% (Figure 5B). There was a higher accumulation of miRNA with 52% GC as observed previously, however, in HYL1-silenced leaves there was >5-fold reduction in the accumulation of this miRNA (Figure 5B). These results suggest that HYL1 is required for the processing of miRNAs with optimum GC both *in vitro* and *in vivo*.

In order to probe if HYL1 has specificity in binding to specific GC containing structures within the precursors, we performed electrophoretic mobility shift assays (EMSA) using three different versions of HYL1, a full-length version (GST-*At*HYL1-6× His), dsRBD12 with two RBDs (6× His dsRBD12), and dsRBD1 alone (Figure 5C). We incubated labelled 21-nt duplex RNAs with different GCs (mature forms of artificial precursor with four stem–loops, Figure 3A), with all three versions of HYL1. Surprisingly, 6хHis dsRBD12 did not bind to any substrate efficiently (data not shown). However, both dsRBD1 as well as GST-*At*HYL1 bound to 52% GC containing duplex RNA much more efficiently than other substrates (Figure 5E). These results clearly indicate the role of HYL1 in specifically selecting stem-loop structures based on their GC composition.

dsRBD1 has higher affinity towards dsRNA and determines substrate specificity (17,69), and it is likely that specific amino acids in this domain might mediate selection of a specific region. We aligned sequences of all 5 DRBs of *A. thaliana* dsRBD1 and that of other homologs such as frog *Xl*RBPA dsRBDs, to identify specific amino acids that might influence specific binding to GC signatures (Supplementary Figure S16D). Several amino acid residues of HYL1 have high specificity for G or C as deduced previously (70). We generated a dsRBD1 peptide, with mutated S18→N, Y23→L residues (Supplementary Figure S16C, Figure 5C) and performed EMSA with four different 21-nt duplex RNA with different GC contents (Figure 5E). The mutant peptide had almost negligible binding when compared to the WT peptide (Figure 5E).

In order to further verify that the mutant lacks the ability to bind to specific GC containing structures, we performed binding assays with miR528 precursor that has 52% GC. As observed in Figure 5F, the WT peptide bound to pre-miR528 structure efficiently while mutations abolished its binding. Due to the longer substrate, the RNA–peptide complex did not enter the gel (Figure 5F). Together, these results indicate that HYL1 is likely to mediate the selection of a specific region in the pri-miRNA transcript for precise dicing by DCL1 (Figure 5G).

### Improvement of artificial miRNAs

In addition to the use of RNA interference in multiple ways, such as by overexpressing antisense RNAs and inverted repeats, specific silencing of targets is achieved in plants through artificial (a)miRNAs. This popular method is capable of targeting a specific member of a large family through the expression of amiR by adopting target rules of a typical miRNA (42,71). However, several candidates of amiRs do not work efficiently, either due to incorrect processing or through inefficient targeting. Multiple laboratories, therefore, optimize more than one amiR candidate to achieve the required knockdown (71–73). We hypothesized that if amiRs can incorporate GC content and signature, they might be more effective in targeting.

**Figure 6.**
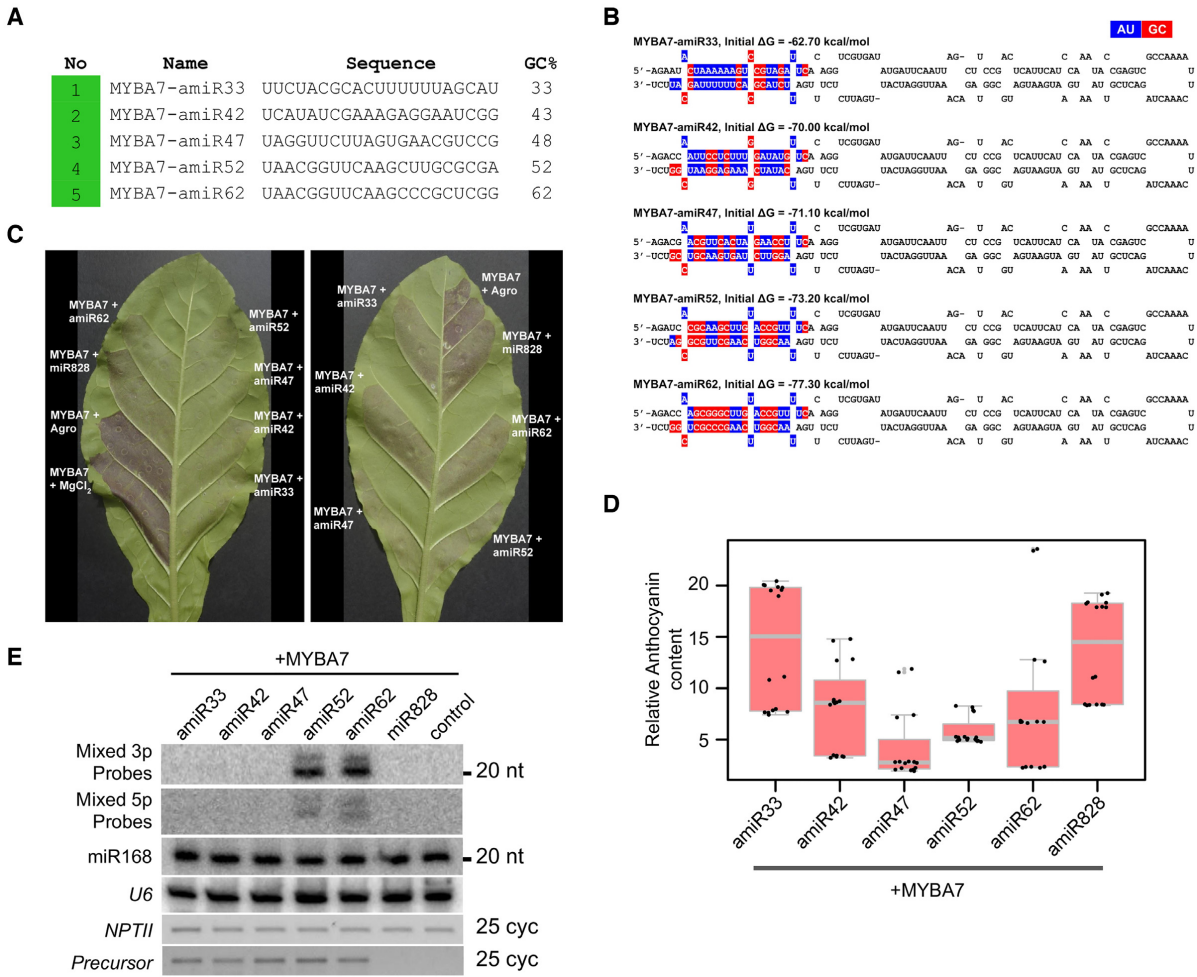
Incorporation of GC signature improves the efficiency of amiRs. (**A**) A selected list of amiR candidates proposed by WMD3 to target *Vv*MYBA7. Colour coded according to WMD3 output. (**B**) Predicted 2D structure of selected amiR precursors. amiR sequence (3p) and its complementary region (5p) are highlighted in colour. (**C**) Photographs of *N. tabacum* leaves co-infiltrated with *Vv*MYBA7 and amiRs. A reduction in the colour is a measure of the efficiency of silencing. (**D**) Anthocyanin content estimation in amiR-infiltrated tissues. This experiment was performed at least three times. (**E**) Northern analysis for the abundance of amiRs (3p sequences) and their stars (5p sequences). *U6* and miR168 served as loading controls. RT-PCR to indicate equal expression of amiR precursor is shown. *NPTII* served as a loading control.

Through the WMD3 online tool, we generated a list of potential candidates to target *Vitis vinifera* MYBA7. *Vv*MYBA7 is a transcription factor capable of inducing the production of anthocyanins upon its expression (74). Among the amiR candidates suggested by WMD3 tool (42), many had GC at either <47% or at higher levels (Supplementary Figure S17A). We selected five different amiRs varying in GC content and signature from the best possible candidates (usually colour-coded in green) (Figures 6A and B, Supplementary Figure S17B and C). We also used Vvi-miR828a, that targets MYBA7 in grapes and other plants as a control (74). miR828 accumulates to lower levels likely due to low GC content of 33%. Three days post-infiltration of *Vv*MYBA7, with and without amiRs, abundant amiRs were produced in candidates having GC signature, while candidates with reduced GC at 33% did not accumulate at optimal levels (Figure 6E). Correspondingly, there was a significant reduction of MYBA7 RNA and anthocyanins in tissues infiltrated with amiRs having optimal GC content and signature matching our previous observations (Figures 6C, D and Supplementary Figure S17D). Although amiR33 mediated silencing was less effective in targeting MYBA7 as seen from anthocyanin accumulation as expected, expression of MYBA7 RNA was unexpectedly lower in co-infiltrated tissues. On the other hand, amiR47 did not accumulate at high levels, while surprisingly targeting MYBA7 effectively as seen in RT-PCR as well as a reduction in anthocyanin accumulation. Expression of transgenes in all infiltrated tissues was at comparable levels as seen from *nptII* expression (Figure 6E). Although we are unable to explain these imperfect correlations, it is possible that variable targeting abilities, localization of RNA molecules, the stability of RNAs as well as target accessibility might have played a role. Similar results were also observed previously (71).

We also used GFP as a target to validate the improvement of amiR technology (Supplementary Figures S18). We selected six candidates with varying GC content and signature (Supplementary Figures S18B and 19A). Targeting sites of these amiRs on GFP were distributed mostly in the second half of the transcript (Supplementary Figures S18B and C). Predicted secondary structures of these precursors were as expected (Supplementary Figure S19B). Three days after the co-infiltration of GFP target with individual amiRs, levels of processed amiRs and target RNAs were analyzed (Supplementary Figures 19D and E). The amiR47, amiRscr47 and amiR62 accumulated comparable levels of miRNAs while others were very low or processed at different sizes (Supplementary Figure S19E). While amiR38 did not accumulate at higher levels in the northern analysis as expected, it was surprisingly efficient in targeting GFP, as seen from reduced GFP RNA and protein accumulation. The target RNA and protein levels were low in amiR47 infiltrated leaves, where the amiR has the optimal GC content of ∼50% and preferred GC signature (Supplementary Figure S19D). These results indicate that incorporation of specific GC signatures in amiR technology has the ability to improve miRNA biogenesis, thereby mediating efficient RNA targeting.

## DISCUSSION

Biogenesis of plant miRNAs has been extensively studied. Most regulators of this pathway have been identified and their functions studied using genetic studies. Based on the structure-function studies, there is sufficient information on specific stages of miRNA biogenesis. However, it is not known how exactly one RNA motif/region is preferred over others for the DCL1 complex to process further. In animals, miRNA biogenesis requires a set of determinants that have been relatively well-characterized (75). For example, U bias in position 1, C or G in position 19 and A or U in position 10 (76). These signatures match well with plant miRNA signatures. However, unlike animal miRNAs, plant miRNAs do not show AU richness in positions 1–7. Since animal miRNA biogenesis is different from plants in multiple ways, it is not possible to apply all these rules for plant miRNA biogenesis.

Based on our data and that of others, it is possible to provide further insights into miRNA selection from long dsRNA. The presence of GC signature that we validated in this report corroborates well with previous observations (40,59). It is not surprising that HYL1 is involved in such a selection; this protein was indeed implicated as a regulator that is required for accurate processing. Using the data generated here, we can infer further specificities of such a selection. Using all the constructs generated here and the outcome of processing, we generated a matrix (Supplementary Figure S20) to compare and identify key GC and AU signatures within a miRNA. From this analysis, we infer that positions 8–9, 18–19 with G or C are the most important signatures, followed by G at position 6. Similarly, it is essential to have A or U in positions 5, 7 and 10. In all constructs with these signatures, there was abundant production of miRNAs. We also find that GC preference in positions 2–4, though very common, is not stringent. It has been hypothesized that HYL1 may lack sequence specificity, instead it exhibits affinity for specific structures within the precursors (17). It is possible that the specific GC signatures we find in our study might influence the local structure of the precursors to enhance HYL1-binding and selection.

The sequence determinants identified above also correlate strongly with sequence features of target RNAs matching predictions of miRNA-target coevolution. This is strong evidence for the presence of a miRNA sequence feature since the high GC region of hundreds of mRNAs that are targeted by miRNAs complement almost perfectly only in the miRNA target region and not elsewhere. In animals, miRNAs target 3′UTR sequences of RNAs through a short seed region that determines silencing, this, in turn, drives the evolution of 3′UTR variations (77,78). In plants, miRNAs target a near-perfect complementary window within the coding region of mRNAs that in turn drives the co-evolution of miRNA and target genes (62,79). The driver of such co-evolution might be the miRNA sequence signature. However, in most plants, mRNAs routinely exhibit higher GC content than other RNAs, probably as a way to distinguish themselves from foreign elements such as AU-rich transposons. Such a clear distinction is also observed experimentally, for example, GC-rich mRNAs are more stable due to inefficient transgene-silencing when compared to AU-rich mRNAs (61). It is interesting to speculate that perhaps only miRNAs, but not siRNAs, have evolved to target high GC-rich sequences such as mRNAs.

Signatures of miRNAs are much more different from their precursors. As observed previously (80), plant miRNA precursors have a higher percentage of AU bias that is absent in other non-coding RNAs. This matches with the prediction that miRNA selection from their precursor is more stringent and might have acted as a strong determinant. It has been predicted that plant miRNAs evolved from inverted duplication of target regions. Selection of mutations to increase AU richness across precursors, but not in mature miRNA/miRNA* regions, might have contributed to the precise selection of this region for further processing. It will be of immense interest to understand the significance of the sequence determinants observed here.

One possible application of the knowledge gained from this analysis is in the optimization of amiR technology that is extremely useful and popular in plants and animals. The Web-based miRNA designer tool (WMD), which works for a wide range of plant species, allows the design of gene-specific amiR candidates for a given target (42,71). However, WMD often generates hundreds of amiR candidates for each target gene and computationally ranks these candidates by sequence complementarity and hybridization energy (dG) with unknown efficacy. Indeed, the amiRNA–targeting can be affected by numerous factors including the optimal abundance of amiRs. Many candidates suggested by WMD3 tool did not silence targets effectively, suggesting that plant amiR tool has varied success levels for a given target (72). AmiR-mediated targeting is also influenced by environmental and developmental factors that regulate amiR biogenesis among other things (81). These issues have promoted the deployment of screening systems to evaluate a large number of amiRs for their efficiency such as ETPamir systems (72). Li *et al.* (72) suggested additional criteria to select amiRs mostly based on targeting abilities. These include target site within the 5′ 200 nucleotides of CDS, fewer mismatches on specific regions, and with dG_amiR-target_/dG_perfect-match_ above 80%. In animals, artificial miRNAs incorporating UG and CNNC motif at the basal stem region (82) were extremely successful in enhancing biogenesis and knock-down abilities of amiRs (83). Our results strongly suggest that incorporation of specific sequence signatures in amiRs has the potential to improve the efficiency of this useful technology. Using multiple targets that vary in GC content and length, we find that the GC signature and content are important for accurate processing and abundance of plant amiRs. These results could improve plant research and crop engineering by making amiR a more predictable genetic and functional genomic technology.

## DATA AVAILABILITY

The scripts used in this study and sRNA sequencing tracks for artificial miRNA are available at GitLab (https://gitlab.com/anushreen/plant_mirna_analysis). The sRNA data generated in this manuscript have been deposited in the Gene Expression Omnibus (GEO) and can be accessed through GSE99094.

## Supplementary Material

gkaa077_Supplemental_File
