# Supplementary material for: A conserved sequence signature is essential for robust plant miRNA biogenesis
Source: Nucleic Acids Res. 2020 Feb 6;48(6):3103–18. doi: 10.1093/nar/gkaa077 (PMC7102948; doi:10.1093/nar/gkaa077)
Supplement: gkaa077_Supplemental_File [file gkaa077_supplemental_file.pdf]

A conserved sequence signature is essential for robust plant miRNA biogenesis

Supplementary information

Supplementary figures

Figure S1. Mature miRNAs have high GC content compared to their precursors.
Figure S2. Abundant mature miRNAs have optimum GC.
Figure S3. Sequence logos of miRNA and siRNAs
Figure S4. miRNAs have unique GC signature.
Figure S5. Plant miRNAs have unique GC signature.
Figure S6. sRNAs other than miRNAs do not show GC signature.
Figure S7. GC signature is not associated with AGO1 preference.
Figure S8. Processing direction of Pre-miRNAs does not seem to affect GC signature
Figure S9. Conserved miRNAs from various species show GC signature.
Figure S10. Less-conserved miRNAs from various species show weak GC signature.
Figure S11. GC signature is observed only in miRNA region
Figure S12. miRNAs target regions on mRNAs also have specific GC signatures.
Figure S13. sRNAs mapping to artificial precursor in two biological replicates.
Figure S14. Predicted secondary structures of precursors used in the study as predicted by Mfold
Figure S15. Comparative analysis of abundance of miRNAs between WT and *hyl1-2*.
Figure S16. dsRBD1 of HYL1 is distinct from other DRB family members.
Figure S17. Targeting abilities of amiR candidates designed through WMD3 tool to target VvMYBA7.
Figure S18. Targeting abilities of amiR candidates designed through WMD3 tool to target GFP.
Figure S19. Incorporation of GC signature improves efficiency of amiRs.
Figure S20. Artificial miRNAs with GC signature are processed better.

Supplementary Tables

Supplementary Table 1: Unique and abundant small RNA sequences aligning to the artificial precursor
Supplementary Table 2: DNA oligos used in this study
Supplementary Table 3: Peptides used for the EMSA studies
Supplementary Table 4: RNA substrates used for EMSA studies

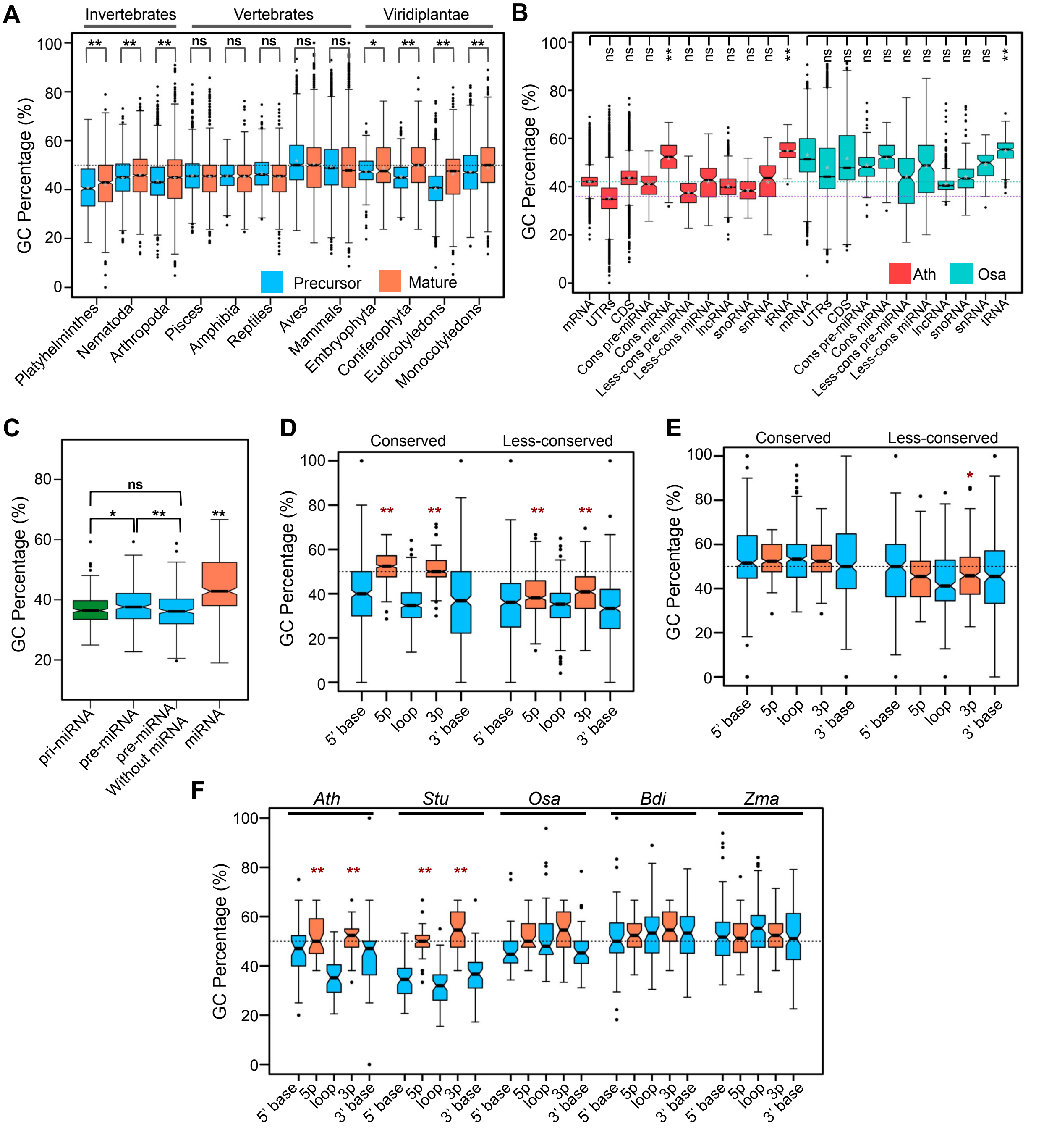

Figure S1. Mature miRNAs have high GC content compared to their precursors.

(A) Boxplots representing GC content of Pre- and mature miRNAs across animal and plant clades. Red asterisk labels significant difference between precursor and mature miRNA GC.

(B) Boxplots representing GC content of various endogenous RNAs from *Arabidopsis* and rice. Grey asterisk indicates mean values. Red asterisk labels significant difference compared to mRNA GC.

(C) Boxplots representing GC content of pri-miRNA, pre-miRNA, pre-miRNA without miRNA region and miRNAs from *Arabidopsis.*

(D) Boxplots representing GC content across different segments of conserved (389) and less-conserved (380) dicot miRNA precursors.

(E) Boxplots representing GC content across different segments of conserved (308) and less-conserved (249) miRNA precursors of monocots. Number of sequences used are given in parenthesis.

(C, D and E) Red asterisk labels significant difference between loop region and mature miRNA GC

(F) Boxplots representing GC content across segments of conserved miRNA precursors that had both 5p and 3p annotated in miRBase v21. Precursors from *A. thaliana* (47), *Solanum tuberosum* (63), *Oryza sativa* (49), *Brachypodium distachyon* (59), *Zea mays* (126) were used.

**P < 0.001, *P < 0.05, Mann-Whitney *U* Test

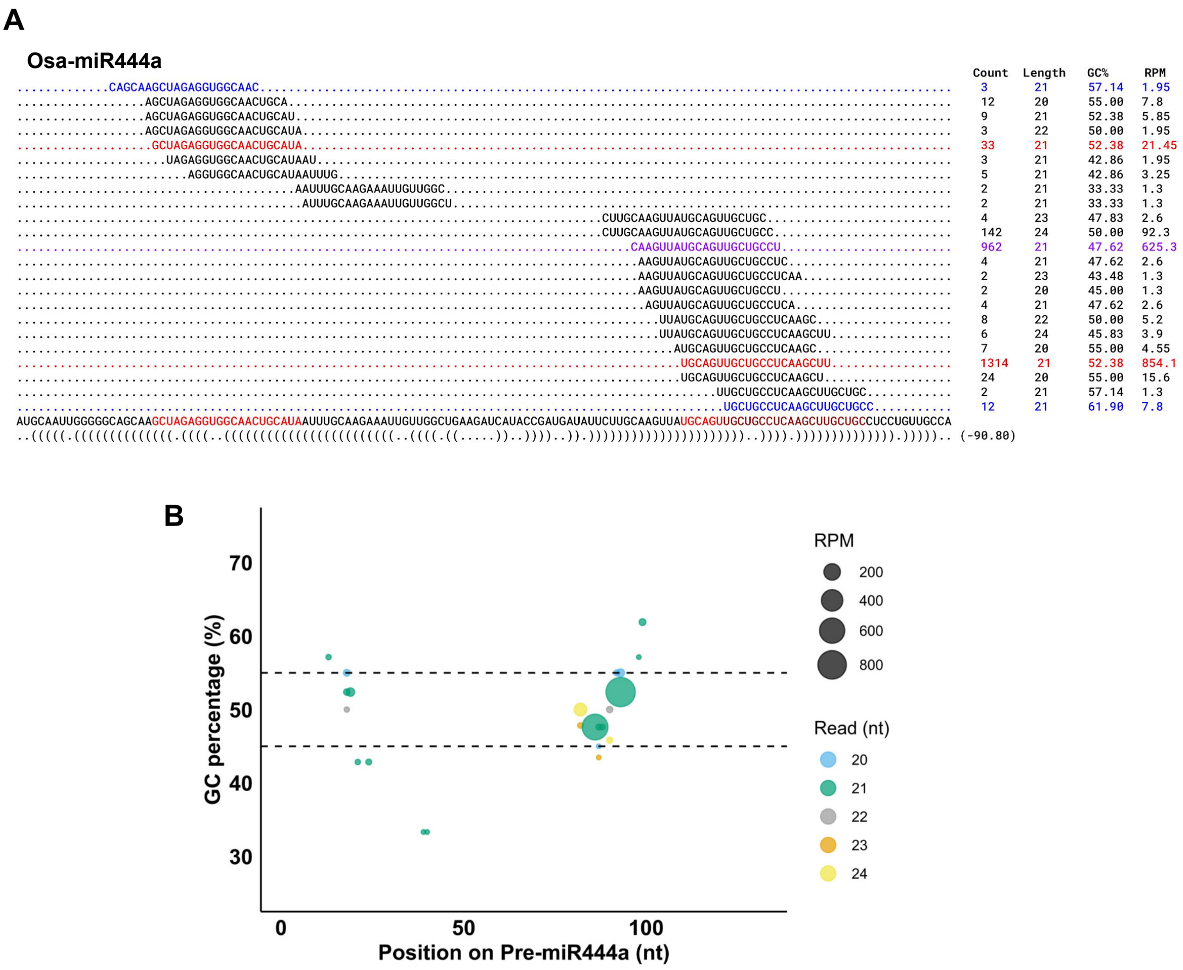

Figure S2. Abundant mature miRNAs have optimum GC.

(A) Abundant miRNA form in a long precursor and a miRNA cluster have 52 % GC. Abundance and GC content of aligned reads (see Methods) are highlighted.

(B) Distribution of abundant reads plotted using data presented in A. Area of circles denotes abundance.

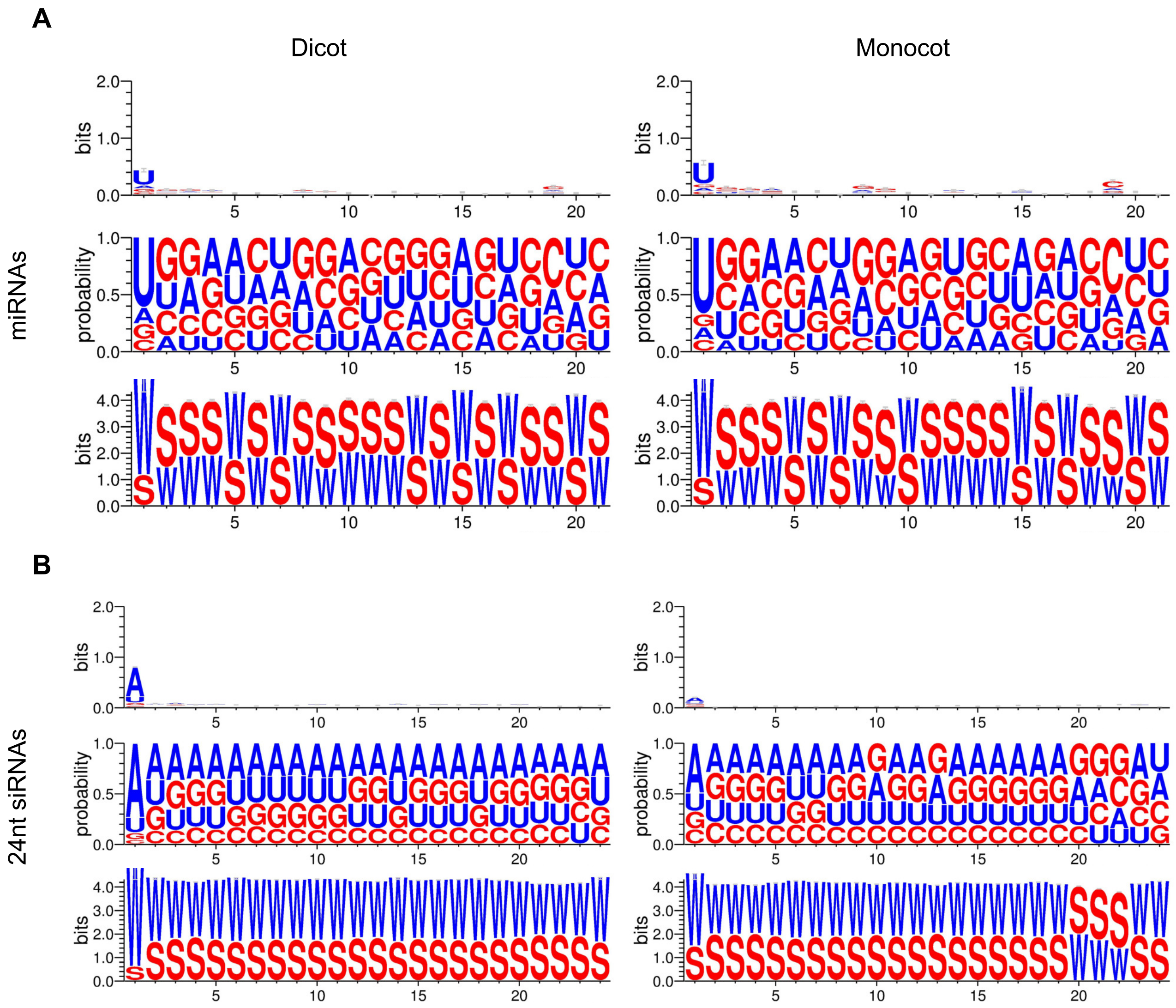

Figure S3. Sequence logos of miRNA and siRNAs

(A) Sequence logos of miRNAs from dicots and monocots, represented as bits, frequency, as well as in SW notation for GC and AU nucleotides, respectively, created using WebLogo 3.

(B) Sequence logos for 24 nt siRNAs.

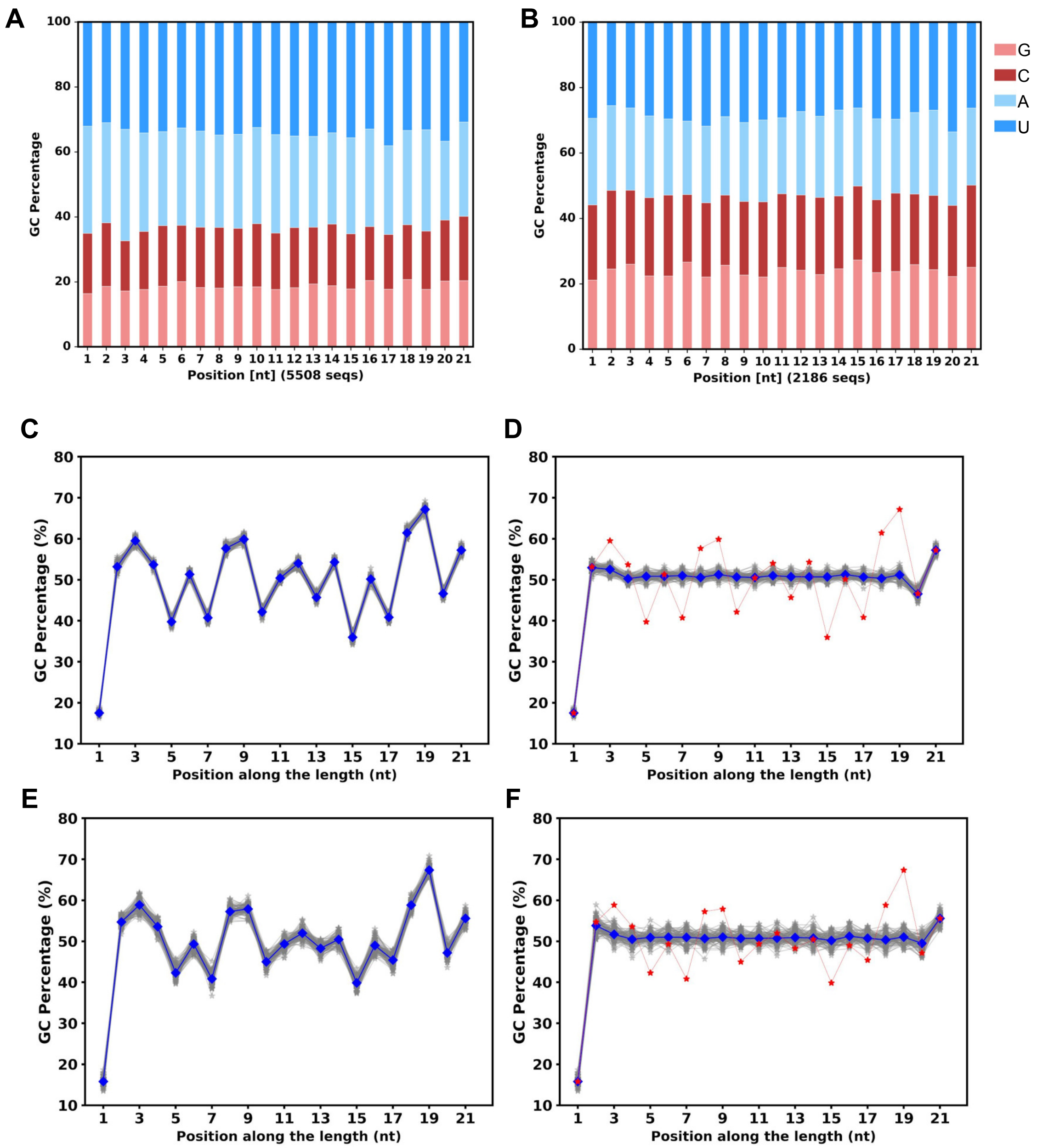

Figure S4. miRNAs have unique GC signature.

(A and B) Position-specific percentage nucleotide abundance of 21-nt sequence adjacent to miRNAs from dicot precursors A, and from monocot precursors B.

(C) Position-specific GC ratio of randomly sampled dicot miRNAs (all 21-nt miRNAs - 8109, sampled 1/3rd of the sequence 500 times). Grey line indicates the GC ratio trend for each sub-sample and blue line indicates mean trend.

(D) For each random sub-sampled dicot miRNAs dinucleotide shuffling was performed (Grey lines) and the mean trend is represented by Blue line. Mean GC trend of random sampling test was overlaid (Red line).

(E) Position-specific GC ratio of randomly sampled monocot miRNAs (all 21-nt miRNAs - 3370, sampled 1/3rd of the sequence 500 times). Grey line indicates the GC ratio trend for each sub-sample and blue line indicates mean trend.

(F) For each random sub-sampled monocot miRNAs dinucleotide shuffling was performed (Grey lines) and the mean trend is represented by Blue line. Mean GC trend of random sampling test was overlaid (Red line).

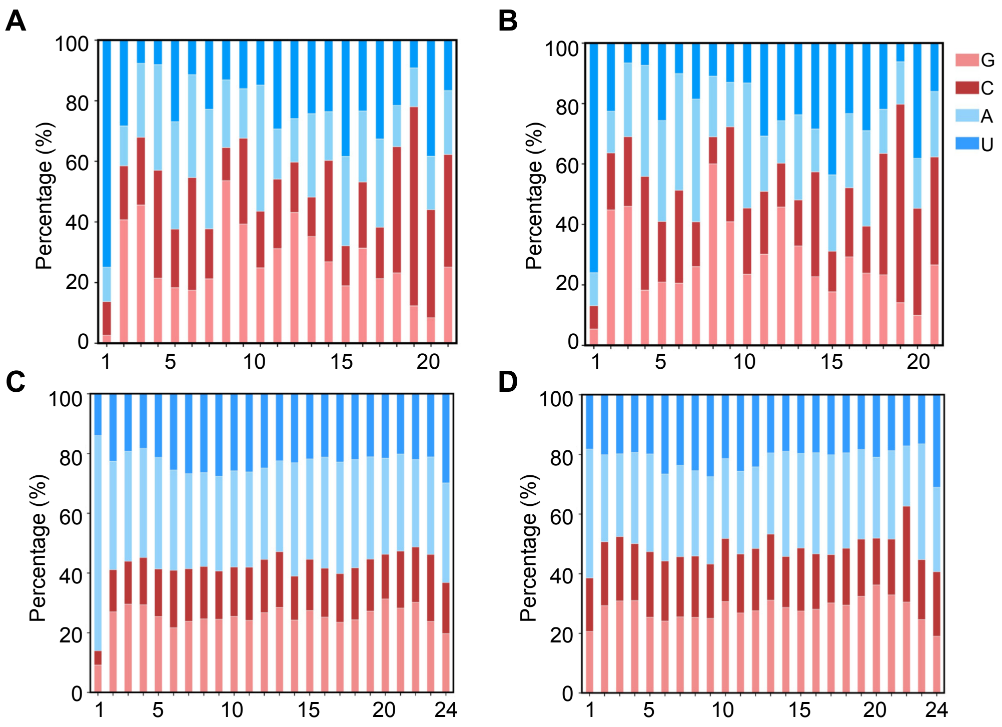

Figure S5. Plant miRNAs have unique GC signature.
(A-D) Position- specific percentage nucleotide abundance of; (A) redundant conserved miRNA sequences from dicots (5250). (B) redundant conserved miRNA sequences from monocots (1759). (C) redundant 24-nt sequences from dicots. (D) redundant 24-nt sequences from monocots.

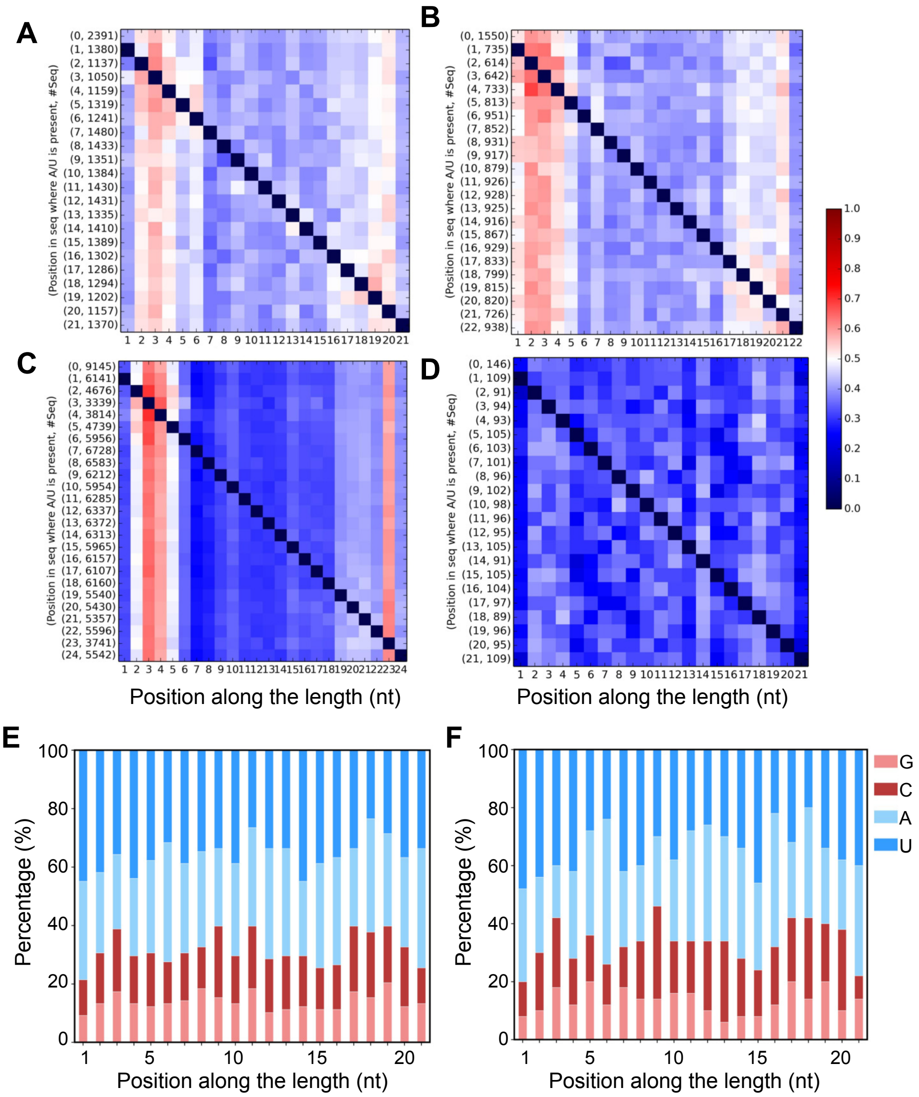

Figure S6. sRNAs other than miRNAs do not show GC signature.
(A) Matrix showing GC signature along abundant 21-nt reads excluding miRNAs from *Arabidopsis*.
(B) GC signature among abundant 22-nt reads excluding miRNAs *Arabidopsis*.
(C) abundant 24-nt reads *Arabidopsis*.
(D) GC signature among all phased siRNAs from *Arabidopsis*. Numbers in parenthesis indicate position of nucleotide and number of reads with A/U in a given position (indicated in dark blue box).
(E) Position-specific percentage nucleotide abundance among unique tasiRNAs (98).
(F) Position-specific percentage nucleotide abundance among abundant phased siRNAs from *A. thaliana* TAS loci (50). Number of sequences used are given in parenthesis.

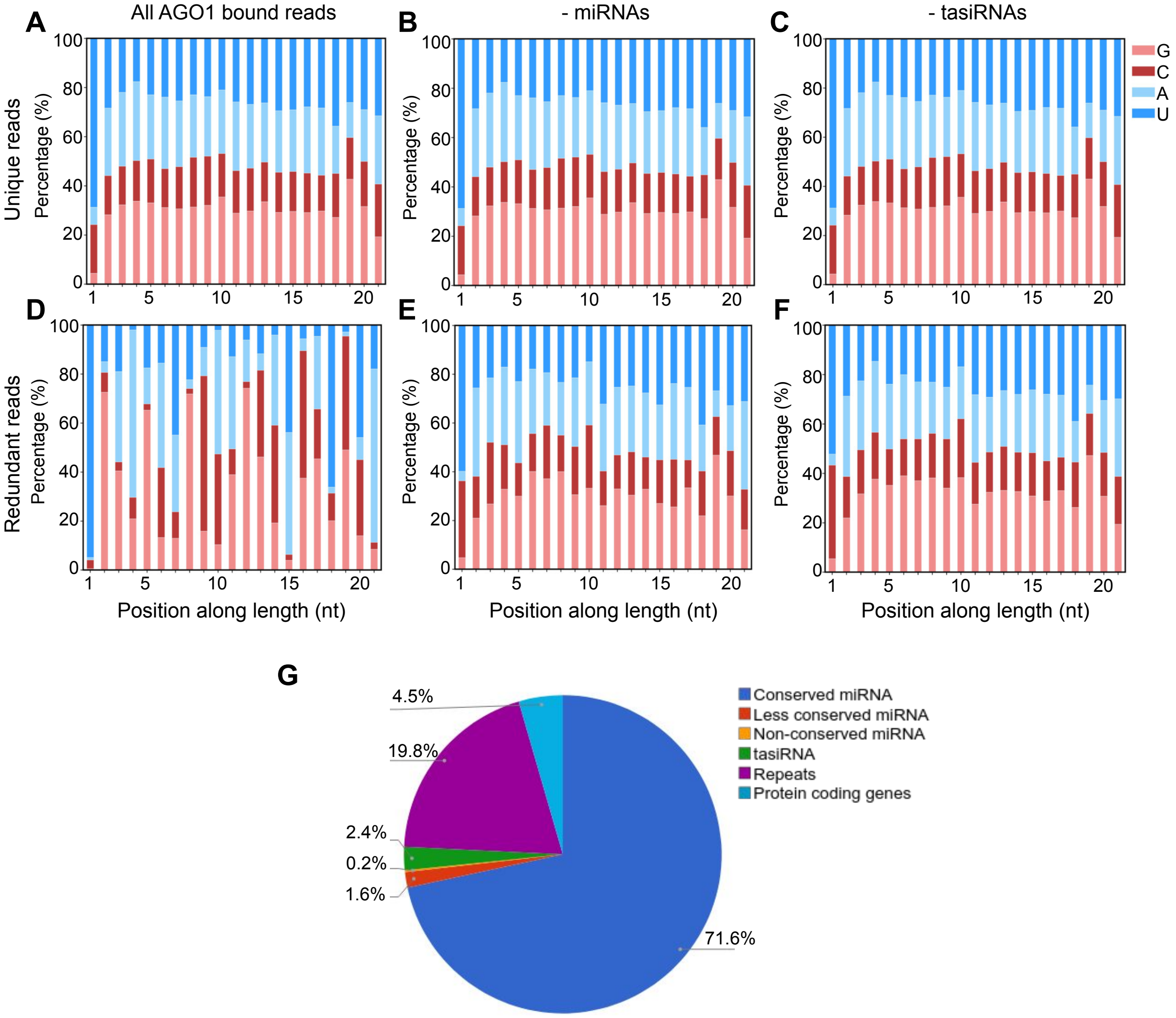

Figure S7. GC signature is not associated with AGO1 preference.

(A-F) Position-specific percentage nucleotide abundance along the length of; (A) all unique sRNAs bound to AGO1, derived from AGO1 IP (21,579). (B) Unique AGO1-bound sRNAs excluding miRNAs (21,366). (C) Unique AGO1-bound sRNAs excluding miRNAs and tasiRNAs (21,211). (D), All redundant AGO1-bound sRNAs (1,368,943). (E) redundant AGO1-bound sRNAs excluding miRNAs (142,143). (F) Redundant AGO1-bound sRNAs excluding miRNAs and tasiRNAs (115,707).

(G) Pie chart showing origin of AGO1-bound sRNAs reanalysed from GSE10036.

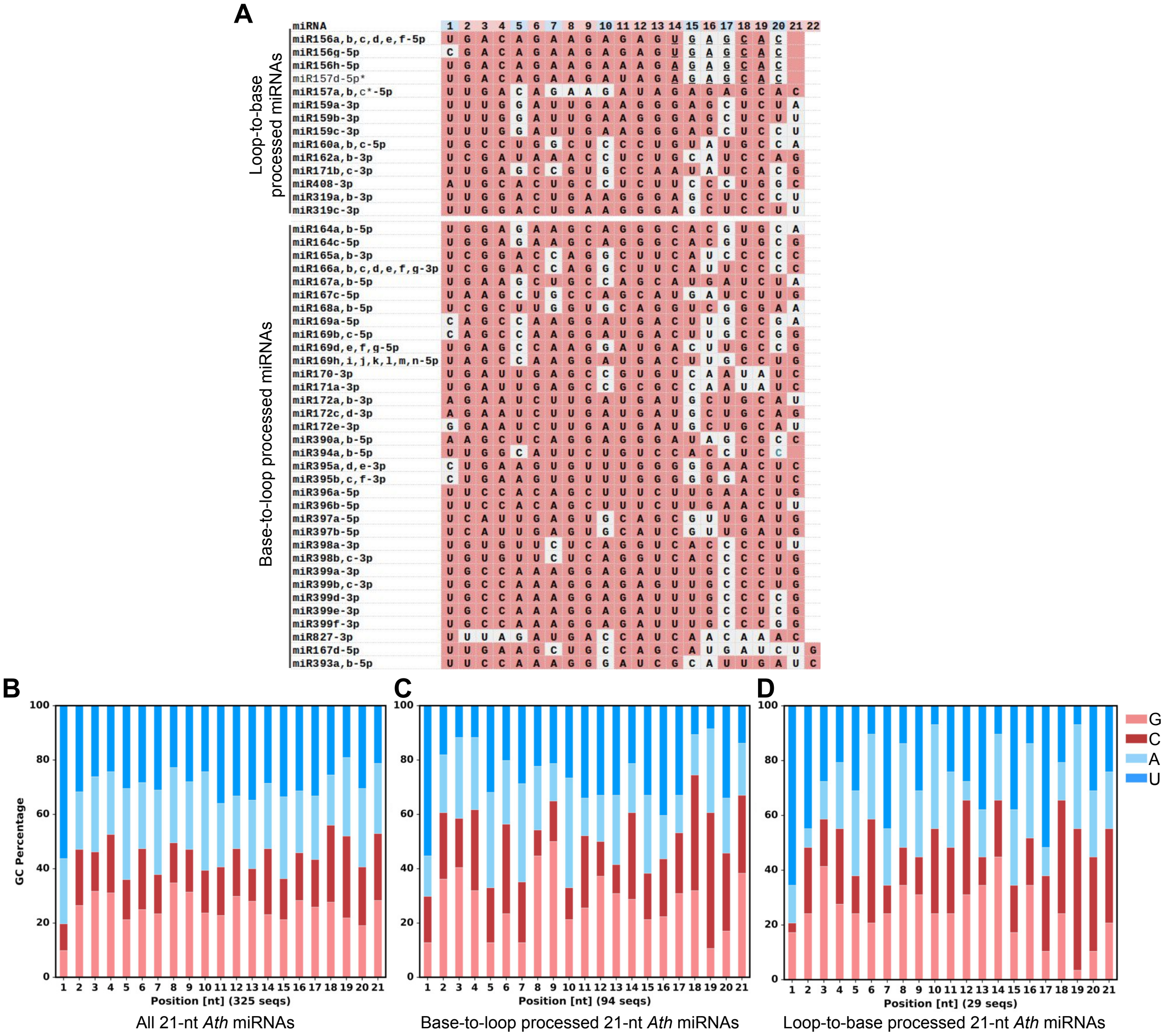

Figure S8. Processing direction of Pre-miRNAs does not seem to affect GC signature

(A) Matrix of all conserved *Arabidopsis* miRNAs. All positions that match predicted GC signature are marked red and those that do not match are colored grey. miRNAs are separated according to their processing direction. *indicate those miRNAs whose processing direction is unclear/ambiguous.

(B) Position-specific percentage nucleotide abundance of all 21-nt *Arabidopsis* miRNAs from miRBase v22.

(C) Position-specific percentage nucleotide abundance of all 21-nt *Arabidopsis* miRNAs that are processed in Base-to-loop direction.

(D) Position-specific percentage nucleotide abundance of all 21-nt *Arabidopsis* miRNAs that are processed in loop-to-base direction.

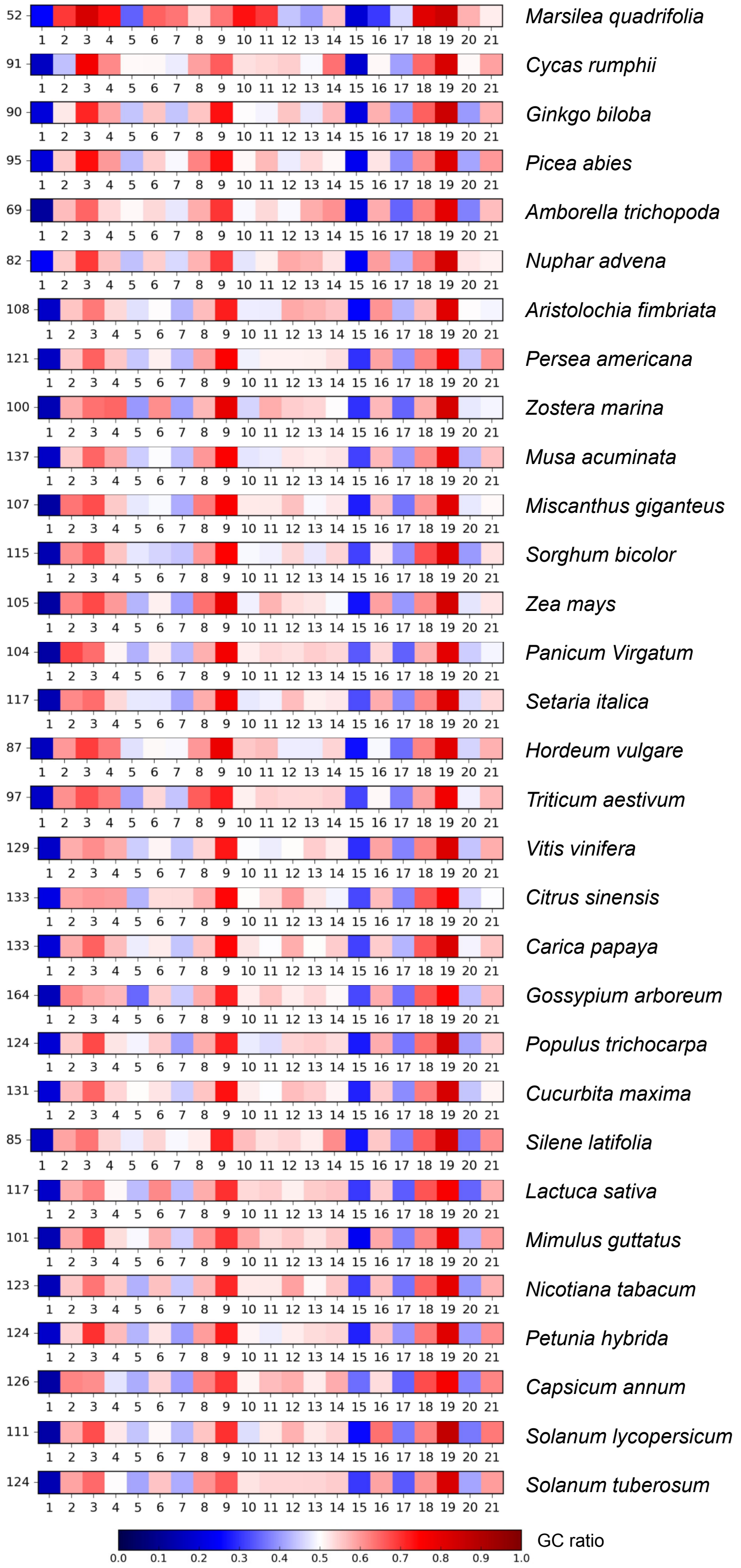

Figure S9. Conserved miRNAs from various species show GC signature.
The colour represents the GC ratio at each position, blue color indicates AU rich positions and red indicates GC rich positions. The numbers on ‘Y’ axis represents number of non-redundant sequences considered per species.

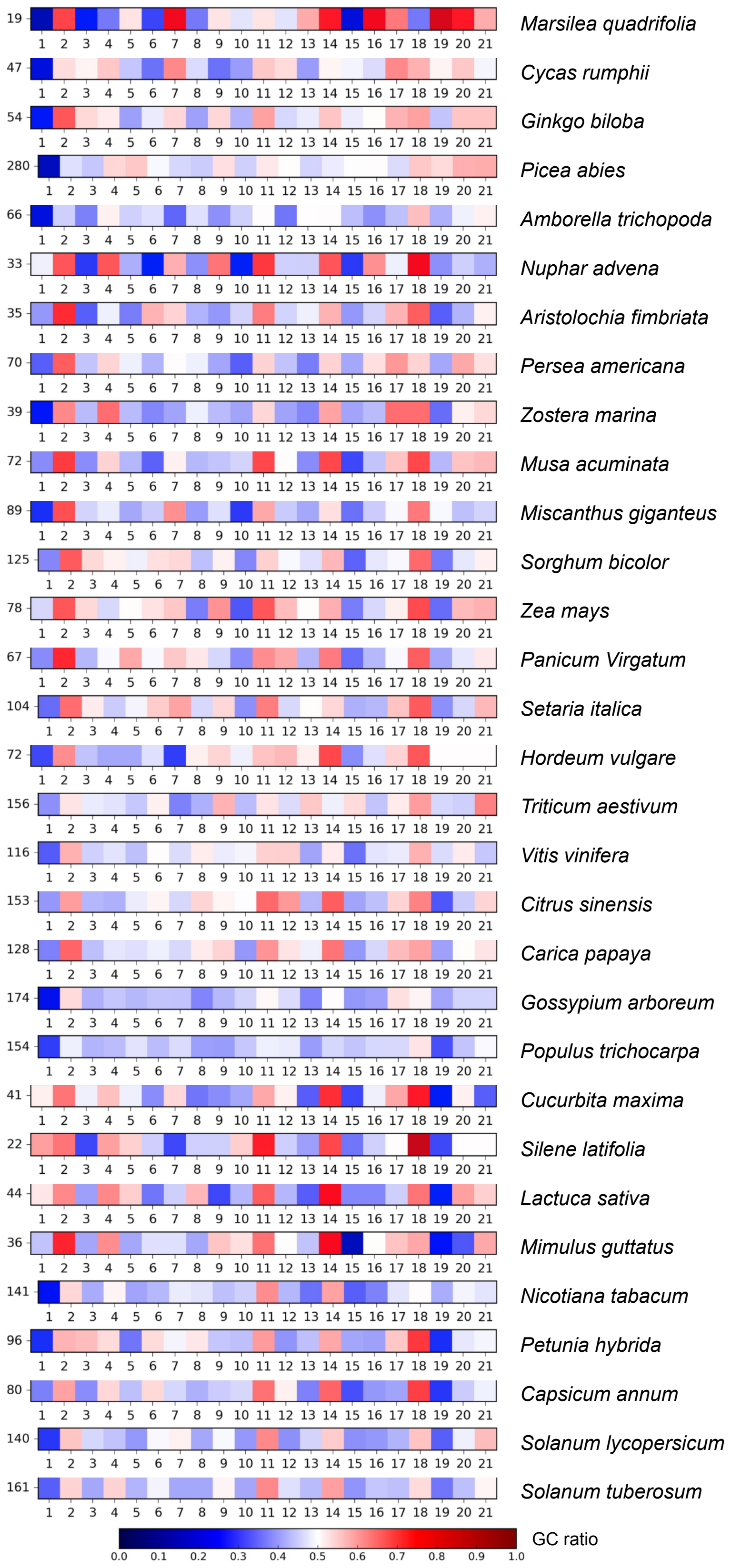

Figure S10. Less-conserved miRNAs from various species show weak GC signature. The colour represents the GC ratio at each position, blue color indicates AU rich positions and red indicates GC rich positions. The numbers on 'Y' axis represents number of non-redundant sequences considered per species.

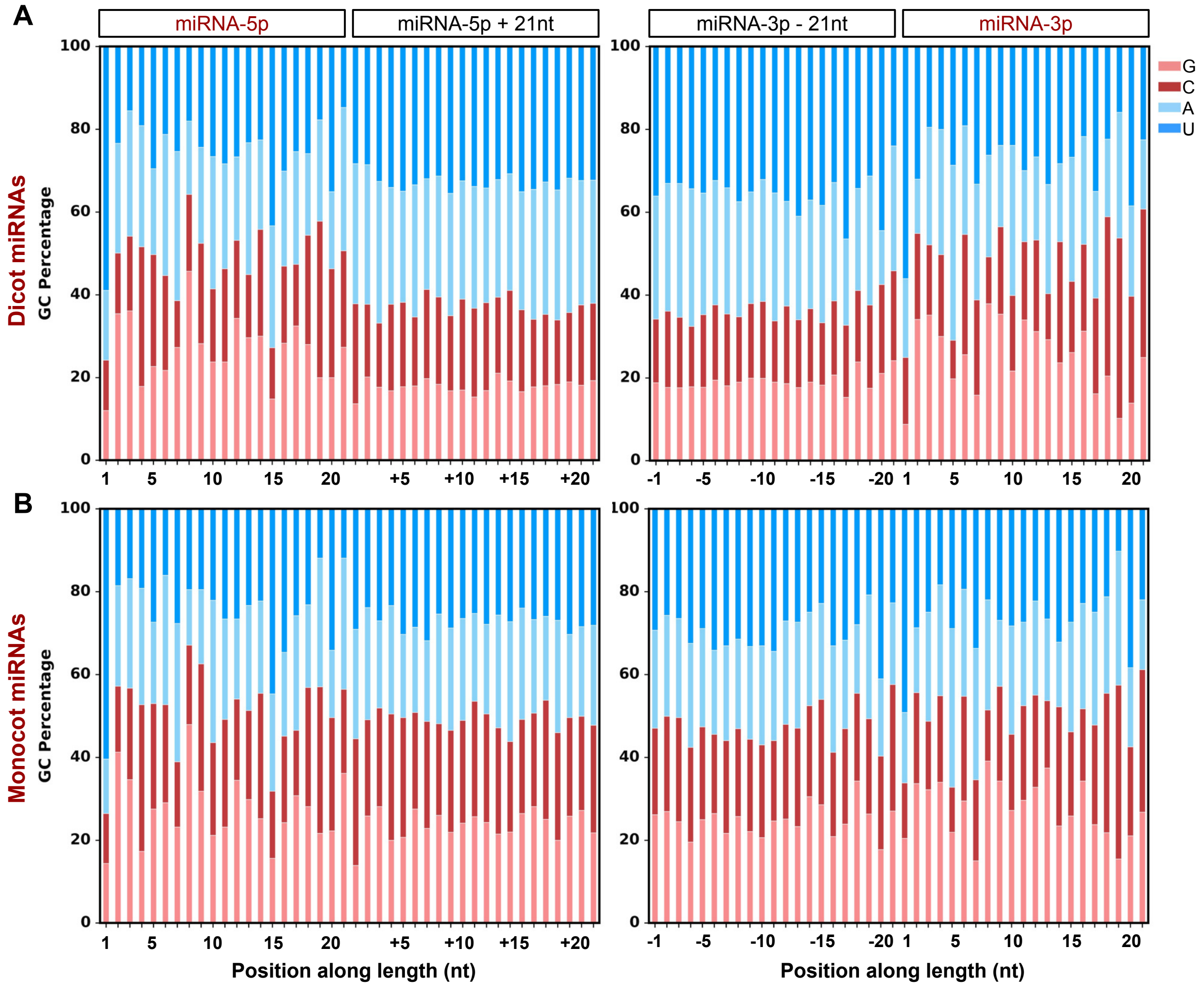

Figure S11. GC signature is observed only in miRNA region

(A and B) Position-specific percentage nucleotide abundance of non-redundant 5p and 3p miRNAs and +21nt or -21 nucleotide window adjacent to them on Pre-miRNA respectively from dicots (~1600 sequences each) B, and from monocots (~650 sequences each) C, from miRBase v22. Number of sequences used are given in parenthesis.

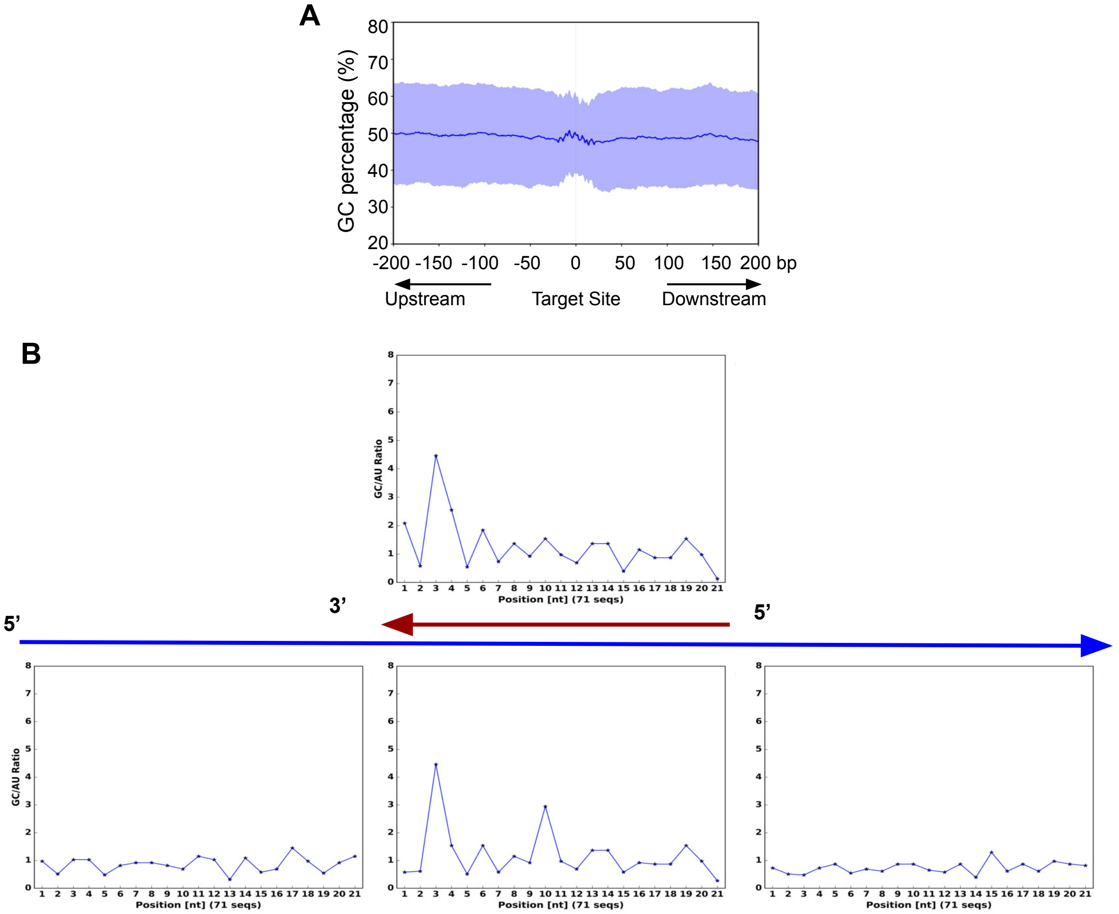

Figure S12. miRNAs target regions on mRNAs also have specific GC signatures.
(A) GC content across miRNA target target representative gene model sequences of *O. sativa* that are predicted by psRNA-Target tool (e-value cutoff of 4, 2453).
(B) Position specific GC/AU ratio of miRNA sequences (upper panel) and target mRNA sequences (lower middle). Adjoining 21-nt windows of miRNA-targeted regions are shown (lower left and lower right).

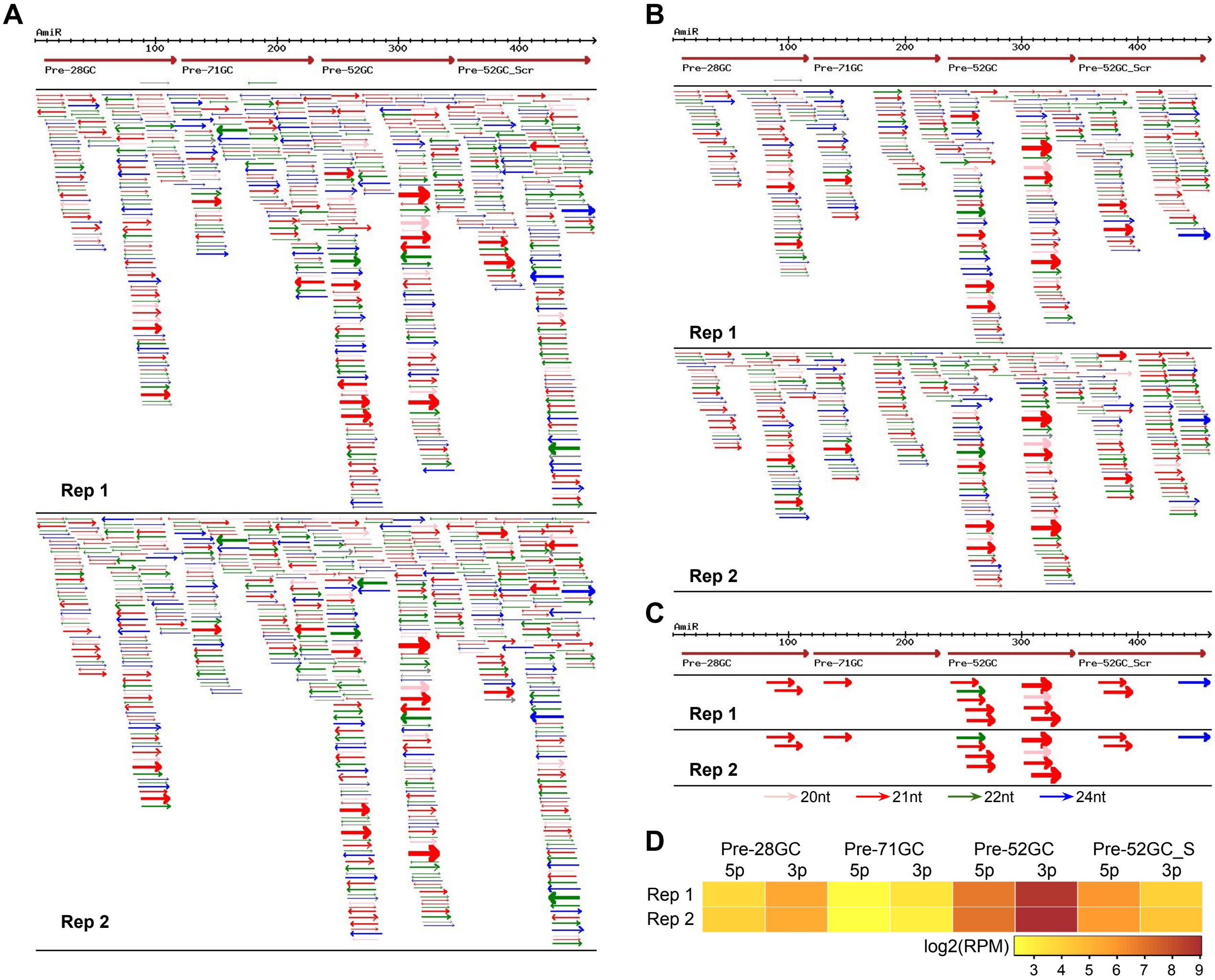

Figure S13. sRNAs mapping to artificial precursor in two biological replicates.
(A) All small RNAs uniquely mapping to artificial precursor with zero mismatch in both and sense and antisense strand in two replicates. Width of the arrows indicates abundance. Length and direction of sRNAs are indicated in colored arrows (pink: 20-nt, red: 21-nt, green: 22-nt, blue: 24-nts). Positions of each stem-loop precursors are marked in brown arrows.
(B) All small RNAs uniquely mapping to artificial precursor with zero mismatch in sense strand.
(C) Small RNAs with abundance of more than 10 RPM in sense strand.
(D) Heatmap showing the abundance of 5p and 3p amiRs from four stemloops.

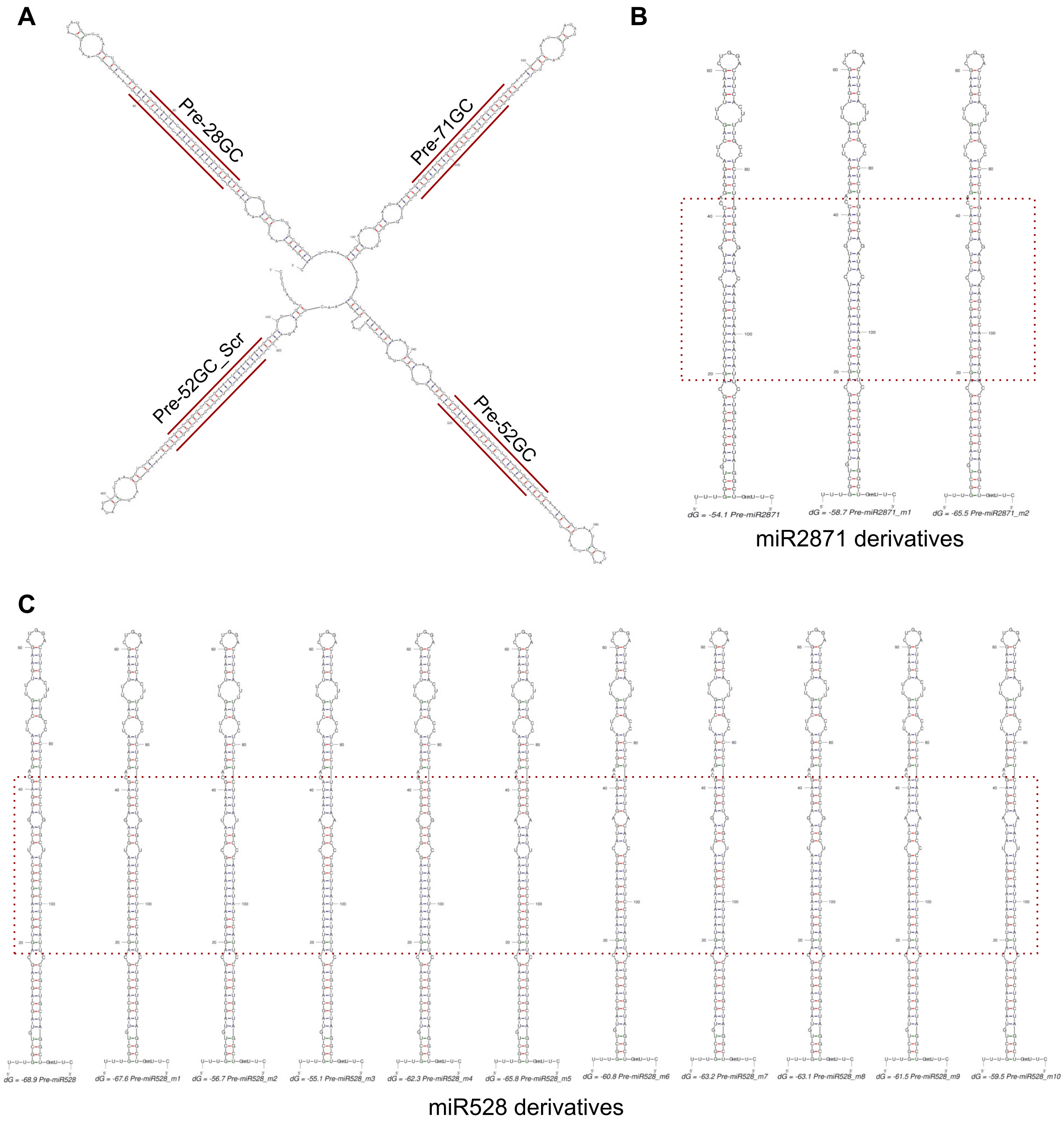

Figure S14. Predicted secondary structures of precursors used in the study as predicted by Mfold

(A) 2D structure of artificial precursor with four stem loops. Highlighted region indicates Rectangle dotted box indicates expected miRNA/miRNA* region for each precursor.

(B) 2D structure of precursors of miR2871 and its derivatives. Dotted rectangle indicates expected miRNA/miRNA* region.

(C) 2D structure of precursors of miR528 and its derivatives. Dotted rectangle indicates expected miRNA/miRNA* region.

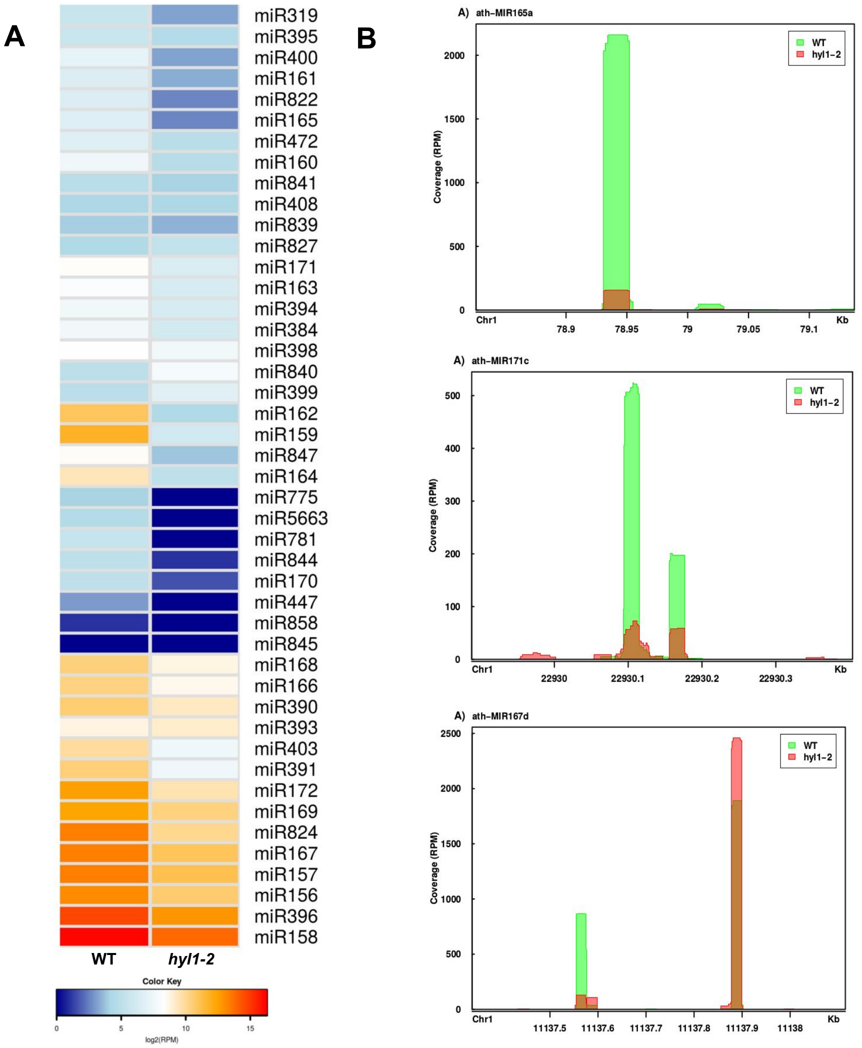

Figure S15. Comparative analysis of abundance of miRNAs between WT and *hyl1-2*.
(A) Heatmap showing reduced abundance of miRNAs in *hyl1-2.* Abundance was calculated using miRProf tool.
(B) Examples of reduced and imprecisely processed miRNAs in *hyl1-2* mutant (red peaks) compared to WT (green peaks).

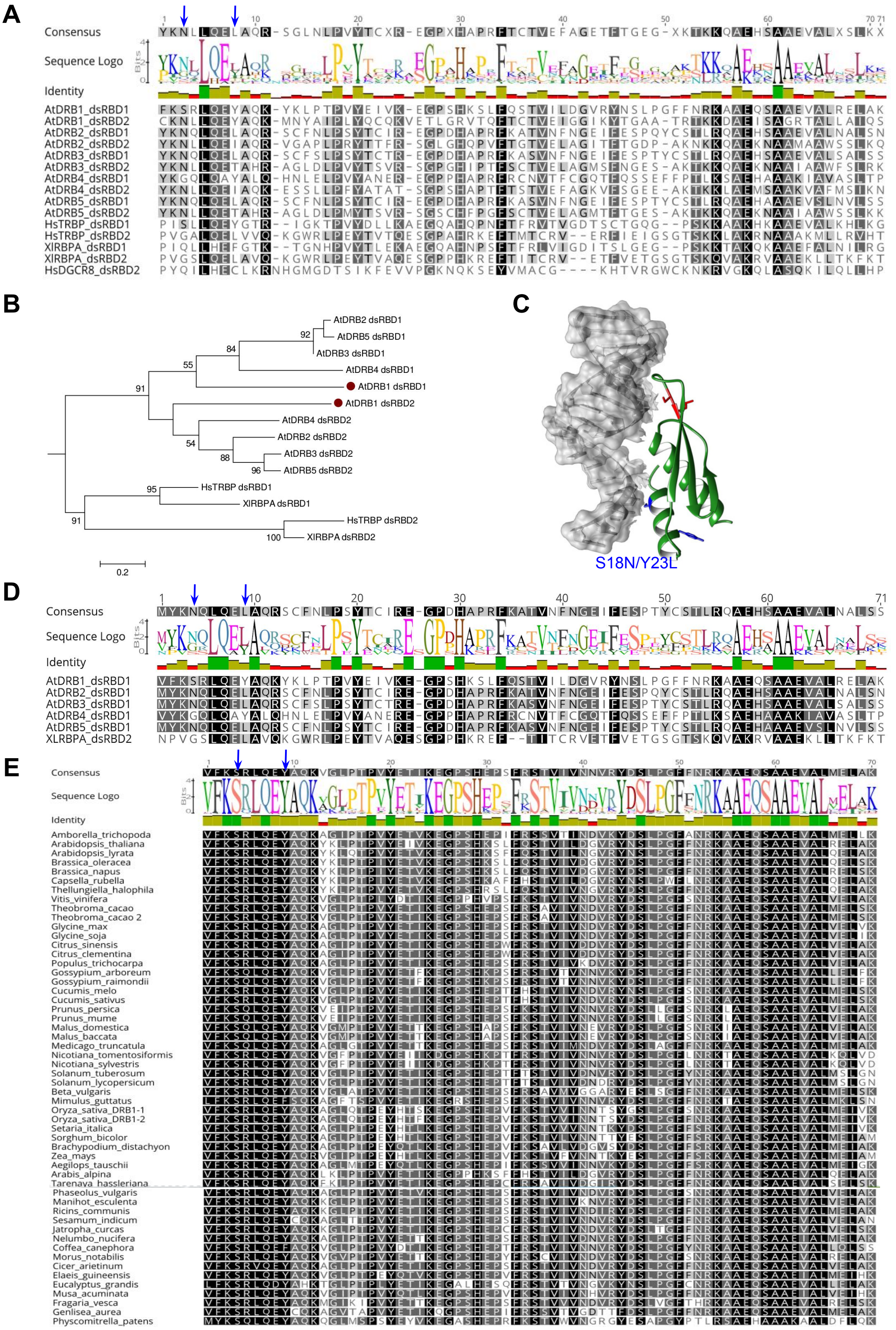

Figure S16. dsRBD1 of HYL1 is distinct from other DRB family members.

(A) Multiple sequence alignment of dsRBDs from all five DRBs (*Arabidopsis)* along with dsRBDs of human TRBP and frog RBPA proteins. Blue arrows indicate mutated residues.

(B) Phylogenetic tree indicating relationship between the candidates used in (A).

(C) Predicted crystal structure of dsRBD1 bound to dsRNA, highlighting the residues interacting with the substrate and those that are mutated.

(D) Multiple sequence alignment of dsRBD1 domain derived from all DRBs of *Arabidopsis.* Conserved residues that are mutated for the study are highlighted.

(E) Multiple sequence alignment of dsRBD1 of HYL1 from diverse plants indicating high sequence conservation among residues unique to HYL1 members.

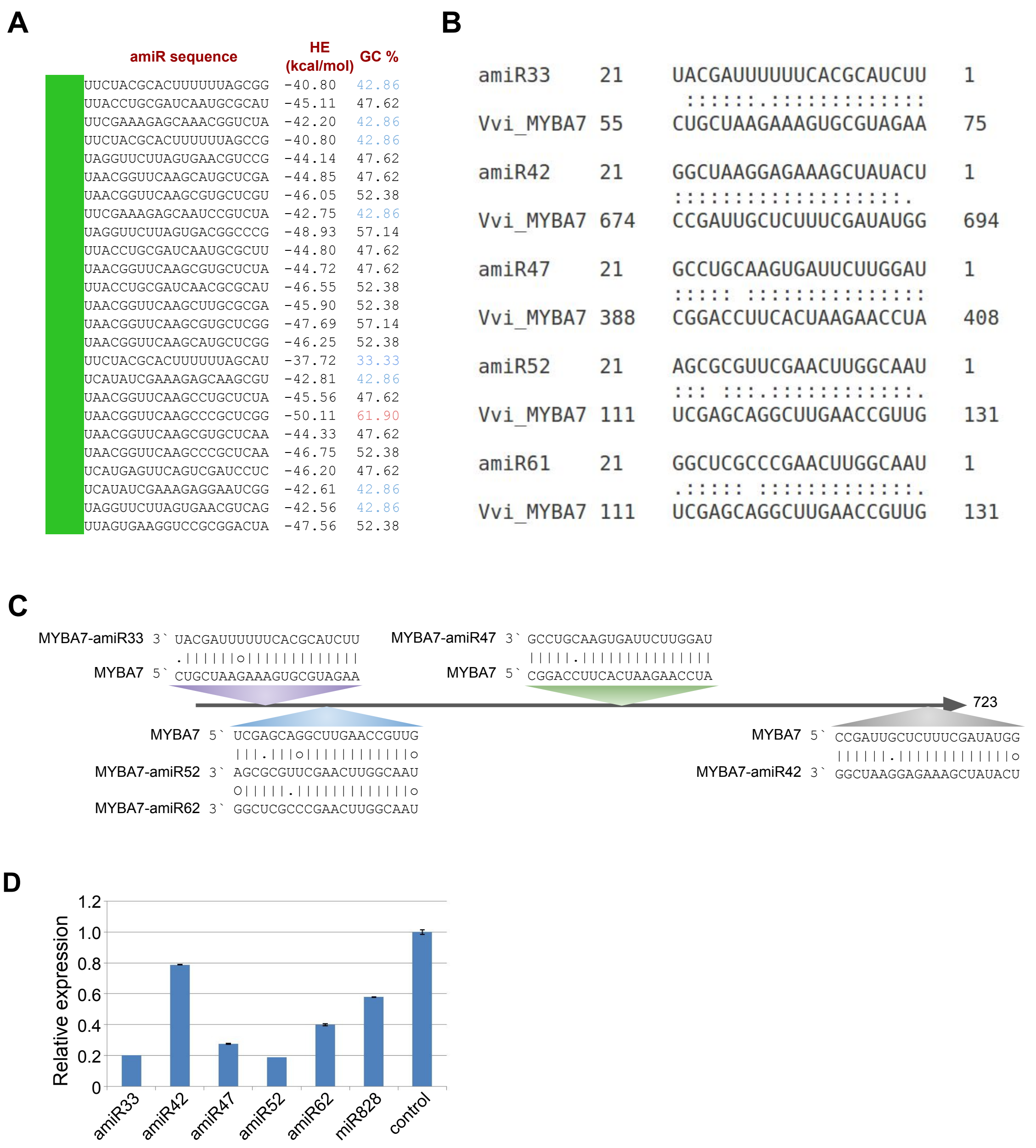

Figure S17. Targeting abilities of amiR candidates designed through WMD3 tool to target VvMYBA7.

(A) List of amiRs obtained by WMD3 against VvMYB7 and their GC content.

(B) Alignment of selected amiR candidates with VvMYBA7.

(C) AmiRs target regions in VvMYBA7 mRNA.

(D) RT-PCR analysis for MYBA7 levels in co-infiltrated leaves.

A

amiR Sequence	HE (kcal/mol)	GC %
UAAAUCGAUUCCCUUAAGCUA	-36.99	33
UAAGUUUUCCGUAUGUCGCAU	-33.3	38
UGUAUUCCAACUUAUGGCCGA	-38.79	42
UGUAUUCCAACUUAUGGCCGG	-38.12	47
UGAUCAGCGAGUUACACGCCG	-43.23	57
UAAAUCGAUUCCCUUACGCUC	-34.39	42
UAAGUUUUCCGUACGUUGCAA	-32.8	38
UGAUCAGCGAGUUACACCCCG	-36.08	57
UAAAUCGAUUCCCUUAAGCUC	-39.07	38
UAAGUUUUCCGUACGUUGCAU	-32.99	38
UGAUCAGCGAGUUACACGCCU	-42.19	52
UUGCACGUAUCCCUUACGCGU	-38.27	52
UAAGAAGUCGUGCCGUUUCUU	-38.77	42
UAUGAUCAGCGAGUUGCGCGC	-45.24	57
UGAUCAGCGAGUUACACGCUG	-40.84	52
UAAGAAGUCGUGCCGUUUCGU	-40.09	47
UUAAAAGGACAGGACUAUCGC	-36.18	42
UUGUCUUGUAGUUACCGUCGG	-36.58	47
UACGUGUCUUGUAGUUCGCGU	-38.66	47
UGUAUUCCAACUUAUGGCCAA	-35.94	38

B

```
amiR33  21  AUCGAAUUCCCUUAGCUAAAU 1
             :::::::::::::::::::
GFP     433 GAGCUUAAGGGAAUCGAUUUC 453

amiR38  21  AACCGGUAUUCAACCUUAUGU 1
            : ::::: :::::::::::::
GFP     473 UCGGCCACAAGUUGGAAUACA 493

amiR47  21  UGCUUUGCCGUGCUGAAGAAU 1
            :.:::.::::::::::::::
GFP     295 AUGAAGCGGCACGACUUCUUC 315

amiRs47 21  UGCGCUUGAUGUUCUGUGCAU 1
            ::: ::::::::::::::::.
GFP     371 ACGGGAACUACAAGACACGUG 391

amiR52  21  GUCGCACAUUGAGCGACUAGU 1
            :.::::: :::::::::::::
GFP     585 CGGCGUGCAACUCGCUGAUCA 605

amiR62  21  GCCGCCGGCCCUAAUGUGUAU 1
            : :: ::.::::::::::::.
GFP     737 CAGCUGCUGGGAUUACACAUG 757
```

C

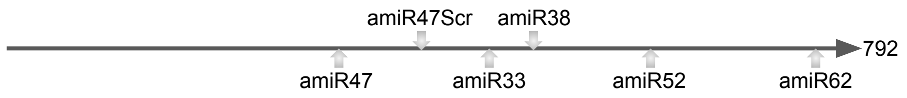

Figure S18. Targeting abilities of amiR candidates designed through WMD3 tool to target GFP.
(A) List of amiRs obtained from WMD3 against GFP and their GC content
(B) Alignment of selected amiR candidates with GFP.
(C) AmiRs target regions in GFP mRNA. Target regions of amiR candidates are colour coded.

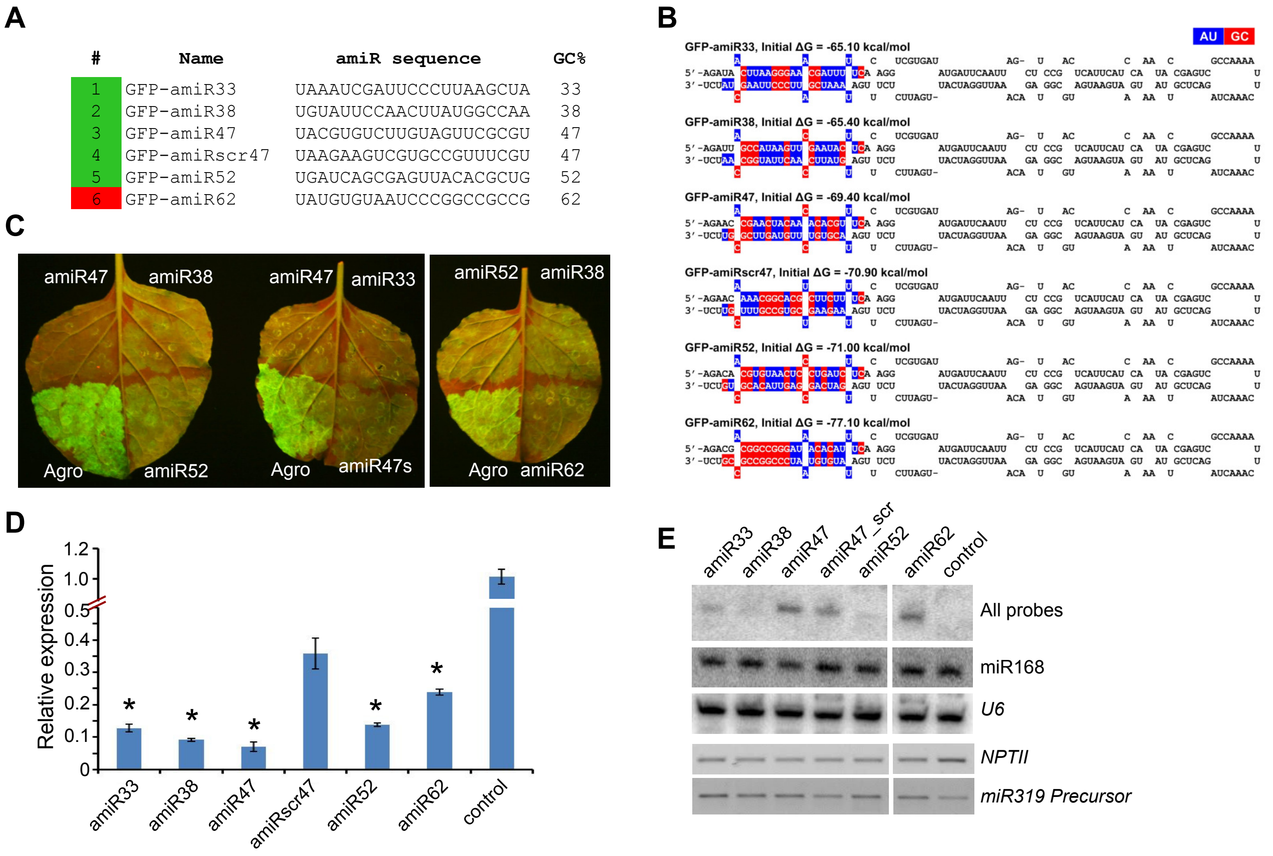

Figure S19. Incorporation of GC signature improves efficiency of amiRs.

(A) Selected list of amiR candidates proposed by WMD3 to target mGFP5. Target score and MFE ratios were calculated by Tapir.

(B) Predicted 2D structure of selected amiR precursors. amiR sequence highlighted in color.

(C) Photographs of tobacco leaves co-infiltrated with 35S:GFP and amiRs. Reduction in GFP fluorescence is an indication for efficiency of silencing.

(D) Real time RT-PCR analysis for GFP in co-infiltrated leaves. Level of GFP normalised to GAPDH and expressed as relative expression compared to control. Error bars indicate standard deviation or three replicates. All amiRs have significantly down regulated GFP in comparison to amiRscr47 (p value < 0.05, t-test).

(E) Northern analysis for the abundance of amiRs. *U6* and miR168 served as loading controls.

amiR name	1	2	3	4	5	6	7	8	9	10	11	12	13	14	15	16	17	18	19	20	21	GC Content	Tapir Score	Tapir ratio	target site	HE (kcal/mol)	Processing efficiency	Targeting efficiency
Osa-miR528	U	G	G	A	A	G	G	G	G	C	A	U	G	C	A	G	A	G	G	A	G	61.90	NA	NA	NA	NA	+++	NA
Osa-miR528_mut1	U	G	G	A	A	G	A	G	G	A	A	U	G	C	A	G	A	G	G	A	G	52.38	NA	NA	NA	NA	+++	NA
Osa-miR528_mut5	U	G	G	C	G	G	G	A	U	A	A	U	A	U	A	A	C	G	G	C	G	52.38	NA	NA	NA	NA	+++	NA
Osa-miR528_mut7	U	A	A	A	U	A	G	G	G	A	A	U	G	C	A	G	A	G	G	A	G	42.86	NA	NA	NA	NA	+++	NA
Osa-miR2871_mut2	U	G	C	U	U	G	A	G	C	U	U	C	U	C	U	G	U	G	C	A	C	52.38	NA	NA	NA	NA	+++	NA
GFP_amir47	U	A	C	G	U	G	U	C	U	U	G	U	A	G	U	U	C	G	C	G	U	47.62	1.5	0.91	295	-38.66	+++	+++
GFP_amirscr47	U	A	A	G	A	A	G	U	C	G	U	G	C	C	G	U	U	U	C	G	U	47.62	2	0.94	371	-40.09	+++	++
GFP_amir62	U	A	U	G	U	G	U	A	A	U	C	C	C	G	G	C	C	G	C	C	G	61.90	3	0.74	737	-35.82	+++	++
MYB_amiR52	U	A	A	C	G	G	U	U	C	A	A	G	C	U	U	G	C	G	C	G	A	52.38	2	0.86	110	-38.63	+++	+++
MYB_amiR62	U	A	A	C	G	G	U	U	C	A	A	G	C	C	C	G	C	U	C	G	G	61.90	2	0.84	110	-40.83	+++	+++
Osa-miR528_mut6	U	A	A	G	G	A	G	A	A	G	G	C	A	U	G	A	G	A	A	G	A	42.86	NA	NA	NA	NA	++	NA
Osa-miR528_mut8	U	G	G	A	A	G	A	A	U	A	A	U	G	C	A	G	A	G	G	A	G	42.86	NA	NA	NA	NA	++	NA
Osa-miR528_mut9	U	G	G	A	A	G	A	G	G	A	G	C	G	C	A	A	U	A	A	U	A	42.86	NA	NA	NA	NA	++	NA
Osa-miR528_mut2	U	G	G	A	U	A	U	A	A	U	G	C	G	C	A	U	U	A	A	A	G	33.33	NA	NA	NA	NA	-	NA
Osa-miR528_mut3	U	A	U	A	A	U	A	A	G	C	G	C	G	C	G	A	U	A	A	U	A	33.33	NA	NA	NA	NA	+	NA
Osa-miR528_mut10	U	G	G	A	A	A	U	G	G	A	A	U	A	U	A	A	U	G	G	A	G	33.33	NA	NA	NA	NA	+	NA
Osa-miR2871	U	A	U	U	U	U	A	G	U	U	U	C	U	A	U	G	G	U	C	A	C	28.57	NA	NA	NA	NA	+	NA
Osa-miR2871_mut1	U	G	C	U	U	U	A	G	U	U	U	C	U	A	U	G	U	G	C	A	C	38.10	NA	NA	NA	NA	++	NA
GFP_amir33	U	A	A	A	U	C	G	A	U	U	C	C	C	U	U	A	A	G	C	U	A	33.33	2	0.87	433	-36.99	++	++
GFP_amir38	U	G	U	A	U	U	C	C	A	A	C	U	U	A	U	G	G	C	C	A	A	38.10	2	0.82	473	-35.94	+	++
GFP_amir52	U	G	A	U	C	A	G	C	G	A	G	U	U	A	C	A	C	G	C	U	G	52.38	1.5	0.90	585	-40.84	+	++
MYB_amiR33	U	U	C	U	A	C	G	C	A	C	U	U	U	U	U	U	A	G	C	A	U	33.33	1.5	1.00	55	-37.12	+	++
MYB_amiR42	U	C	A	U	A	U	C	G	A	A	A	G	A	G	G	A	A	U	C	G	G	42.86	1.5	0.86	673	-36.29	+	++
MYB_amiR47	U	A	G	G	U	U	C	U	U	A	G	U	G	A	A	C	G	U	C	C	G	47.62	1	0.85	387	-37.71	+	+++

Figure S20. Artificial miRNAs with GC signature are processed better.
List of amiRs and their GC signature. Blue colour indicates AU rich positions and red color indicates GC rich positions.

Supplemental table 1 | Unique and abundant small RNA sequences aligning to the artificial precursor

Region	Sequence (5`-3`)	Read Length	Rep1 (RPM)	Rep2 (RPM)	GC %
28miRNA_5p	AAGATACTATACGTTGCAAAG	21	5.2	7.6	33.33
28miRNA_5p	CTCGAATAAGATACTATACGT	21	1.2	3.2	33.33
28miRNA_5p	AATAAGATACTATACGTTGCA	21	1.6	2.4	28.57
28miRNA_5p	AATAAGATACTATACGTTGCAAAG	24	2	0.4	29.17
28miRNA_3p	TATACTATCTTATTCGAGCTT	21	14	16	28.57
28miRNA_3p	GTATACTATCTTATTCGAGCT	21	9.2	9.6	33.33
28miRNA_3p	AACGTATACTATCTTATTCGAGCT	24	3.6	4	33.33
28miRNA_3p	TATACTATCTTATTCGAGCT	20	4	2	30
28miRNA_3p	CGTATACTATCTTATTCGAGC	21	3.2	2	38.1
71miRNA_5p	TCGAACAGGGCGCCAGTGCCC	21	2.8	1.2	71.43
71miRNA_3p	CACTGCCGCCCTGTTCGAGCT	21	4	2.8	66.67
71miRNA_3p	CACTGCCGCCCTGTTCGAGCTT	22	2.4	2	63.64
52miRNA_5p	TCGAACTAGGTCTCAGTGCTC	21	52.4	52.8	52.38
52miRNA_5p	CTCGAACTAGGTCTCAGTGCT	21	37.2	40.4	52.38
52miRNA_5p	AACTAGGTCTCAGTGCTCGCA	21	2	6	52.38
52miRNA_5p	AAGAAGCTCGAACTAGGTCTCAGT	24	5.6	2.8	45.83
52miRNA_5p	TCGAACTAGGTCTCAGTGCT	20	4	4.4	50
52miRNA_5p	TAGGTCTCAGTGCTCGCAAAG	21	4.4	3.6	52.38
52miRNA_5p	CGAACTAGGTCTCAGTGCTCG	21	2	3.6	57.14
52miRNA_5p	AGAAGCTCGAACTAGGTCTCAGTG	24	2.4	0.4	50
52miRNA_3p	CACTGTGACCTAGTTCGAGCT	21	260.8	355.6	52.38
52miRNA_3p	CAGCGAGCACTGTGACCTAG	20	52.8	67.6	60
52miRNA_3p	CAGCGAGCACTGTGACCTAGT	21	47.6	64.4	57.14
52miRNA_3p	AGCACTGTGACCTAGTTCGAG	21	8.8	5.6	52.38
52miRNA_3p	CACTGTGACCTAGTTCGAGCTT	22	6.4	8.4	50
52miRNA_3p	CACTGTGACCTAGTTCGAGC	20	6.4	6	55
52miRNA_3p	GCACTGTGACCTAGTTCGAGC	21	4.4	2.8	57.14
52miRNA_3p	ACTGTGACCTAGTTCGAGCTT	21	2.4	3.6	47.62
52miRNA_3p	AGCGAGCACTGTGACCTAGT	20	3.2	1.6	55
52miRNA_3p	ACTGTGACCTAGTTCGAGCT	20	1.2	2.4	50
ScrmiRNA_5p	ATTTCGGCCCCGGCGGCAAAG	21	38	28	66.67
ScrmiRNA_5p	TATTAATTTCGGCCCCGGCGG	21	13.6	11.2	57.14
ScrmiRNA_5p	TTATTAATTTCGGCCCCGGCG	21	0.8	2.8	52.38

ScrmiRNA_5p	TATTAATTTCGGCCCCGGCGGC	22	1.6	2.4	59.09
ScrmiRNA_5p	CTTATTAATTTCGGCCCCGGC	21	0.8	2	52.38
ScrmiRNA_3p	CAGCCGCCGGGCCCGAAATT	20	5.2	8	70
ScrmiRNA_3p	CAGCCGCCGGGCCCGAAATTA	21	6.8	5.6	66.67

Supplemental table 2 | DNA oligos used in this study

Name	Sequence(5'-3')	Application
Probe1_amiR28	AACGTATAGTATCTTATTCGA	Northern blot
Probe2_amiR71	GGGCACTGGCGCCCTGTTCGA	Northern blot
Probe3_amiR52	GAGCACTGAGACCTAGTTCGA	Northern blot
Probe4_amiR52_scrambled	CGCCGGGGCCGAAATTAATAA	Northern blot
amiR28_AS	GCTCGAATAAGATAGTATACGTT	Northern blot
amiR72_AS	GCTCGAACAGGGCGGCAGTGCCC	Northern blot
amiR52_AS	GCTCGAACTAGGTCACAGTGCTC	Northern blot
amiR52Scr_AS	GCTTATTAATTTCGGGCCCGGCG	Northern blot
miR528_WT_probe	CTCCTCTGCATGCCCCTTCCA	Northern blot
miR528_m1_probe	CTCCTCTGCATTCCTCTTCCA	Northern blot
miR528_m2_probe	CTTTAATGCGCATTATATCCA	Northern blot
miR528_m3_probe	TATTATCGCGCGCTTATTATA	Northern blot
miR528_m4_probe	CGCCGCCGCGCTATTATTATA	Northern blot
miR528_m5_probe	CGCCGTTATATTATCCCGCCA	Northern blot
miR528_m6_probe	TCTTCTCATGCCTTCTCCTTA	Northern blot
miR528_m7_probe	CTCCTCTGCATTCCCTATTTA	Northern blot
miR528_m8_probe	CTCCTCTGCATTATTCTTCCA	Northern blot
miR528_m9_probe	TATTATTGCGCTCCTCTTCCA	Northern blot
miR528_m10_probe	CTCCATTATATTCCATTTCCA	Northern blot
miR528_WT_AS	AATGGAAGAGGCAAGCACAGGAG	Northern blot
miR528_m1_AS	AATGGAAGAGGAAAGCACAGGAG	Northern blot
miR528_m2_AS	AATGGATATAATGGGCAATAAAG	Northern blot
miR528_m3_AS	AATATAATAAGCGGGCGTTAATA	Northern blot
miR528_m4_AS	AATATAATAATAGGGCGCCGGCG	Northern blot
miR528_m5_AS	AATGGCGGGATAAAATATCGGCG	Northern blot
miR528_m6_AS	AATAAGGAGAAGGGATGTGAAGA	Northern blot
miR528_m7_AS	AATAAATAGGGAAAGCACAGGAG	Northern blot
miR528_m8_AS	AATGGAAGAATAAAGCACAGGAG	Northern blot
miR528_m9_AS	AATGGAAGAGGAGGGCATTAATA	Northern blot
miR528_m10_AS	AATGGAAATGGAAAATATTGGAG	Northern blot
miR2871_WT_probe	GTGACCATAGAAACTAAAATA	Northern blot
miR2871_m1_probe	GTGCACATAGAAACTAAAGCA	Northern blot
miR2871_m2_probe	GTGCACAGAGAAGCTCAAGCA	Northern blot
miR2871_WT_AS	AATATTTTAGTTTGTATCGTCAC	Northern blot

miR2871_m1_AS	AATGCTTTAGTTTGTATCTGCAC	Northern blot
miR2871_m2_AS	AATGCTTGAGCTTGTCTCTGCAC	Northern blot
GFP_AmiR33_Probe	TAGCTTAAGGGAATCGATTTA	Northern blot
GFP_AmiR38_Probe	TTGGCCATAAGTTGGAATACA	Northern blot
GFP_AmiR47_Probe	ACGAAACGGCACGACTTCTTA	Northern blot
GFP_AmiRscr47_Probe	ACGCGAACTACAAGACACGTA	Northern blot
GFP_AmiR52_Probe	CAGCGTGTAACTCGCTGATCA	Northern blot
GFP_AmiR57_Probe	GCGCGCAACTCGCTGATCATA	Northern blot
GFP_AmiR62_Probe	CGGCGGCCGGGATTACACATA	Northern blot
GFP-amiR33_AS	GAAAAATCGTTTCCCTTAAGTTA	Northern blot
GFP-amiR38_AS	GAAGTATTCGAACTTATGGCTAA	Northern blot
GFP-amiR47_AS	GAAACGTGTGTTGTAGTTCGTGT	Northern blot
GFP-amiR47Scr_AS	GAAAAGAAGACGTGCCGTTTTGT	Northern blot
GFP-amiR52_AS	GAAGATCAGGGAGTTACACGTTG	Northern blot
GFP-amiR57_AS	GAAATGATCTGCGAGTTGCGTGC	Northern blot
GFP-amiR62_AS	GAAATGTGTTATCCCGGCCGTCG	Northern blot
MYBA7_AmiR33_Probe	ATGCTAAAAAAGTGCGTAGAA	Northern blot
MYBA7_AmiR42_Probe	CCGATTCCTCTTTCGATATGA	Northern blot
MYBA7_AmiR47_Probe	CGGACGTTCACTAAGAACCTA	Northern blot
MYBA7_AmiR52_Probe	TCGCGCAAGCTTGAACCGTTA	Northern blot
MYBA7_AmiR62_Probe	CCGAGCGGGCTTGAACCGTTA	Northern blot
MYB-amiR33_AS	GAATCTACGGACTTTTTTAGTAT	Northern blot
MYB-amiR42_AS	GAACATATCCAAAGAGGAATTGG	Northern blot
MYB-amiR47_AS	GAAAGGTTCATAGTGAACGTTCG	Northern blot
MYB-amiR52_AS	GAAAACGGTACAAGCTTGCGTGA	Northern blot
MYB-amiR62_AS	GAAAACGGTACAAGCCCGCTTGG	Northern blot
amiR-HYL1_probe	GAGAGCCTCCAAGGTATGTCA	Northern blot
Pre-miR528_F	GATCCCAGCAGCAGCCACAGC	RT-PCR
Pre-miR528_R	GCTCGCTGCTGATGCTGATGC	RT-PCR
AtDRB1_FWD	CTCGGATCCATGACCTCCACTGATGTTTC	PCR amplification of AtHYL1
AtDRB1_REV_6xHIS_SacI	TTGGAGCTCTTAGTGATGGTGATGGTGATGTGC GTGGCTTGCTTC	PCR amplification of AtHYL1
NtHYL1_SacI_F	ATATTGAGCTCGTGTGCAGCGACGATACC	Antisense NtHYL1 PCR
NtHYL1_BamHI_R	ACTGGGATCCTTTCATATTGTGGAATTGC	Antisense NtHYL1 PCR
Vvi-MYBA7_FWD	GGGCTCTTTAGGTCTGCGG	RT-PCR
Vvi-MYBA7_REV	TCTACCTGCGATCAATGCCC	RT-PCR
GFP_FWD	ACCTGTCCACACAATCTGCCC	RT-PCR
GFP_REV	GAGCTCTTATTTGTATAGTTCATCC	RTPCR
NPTII_FWD	GAATGAACTGCAGGACGAGG	RT-PCR

NPTII_REV	ATCGACAAGACCGGCTTCCA	RT-PCR
NtGAPDH_FWD	GGAGGAGGGAACAACAAGAGG	RT-PCR
NtGAPDH_REV	AGATGCCGTCAGTGCCGA	RT-PCR

Supplemental table 3 | Peptides used for the EMSA studies

Name	Sequence	Supplier
AtHYL1-dsRBD1_WT	VFKSRLQEYAQKYKLPTPVYEIVKEGPSHKSLFQSTVILDGVR YNSLPGFFNRKAAEQSAAEVALRELAK	LifeTein, USA
AtHYL1-dsRBD1$^{S18N/Y23L}$	VFKNRLQELAQKYKLPTPVYEIVKEGPSHKSLFQSTVILDGVR YNSLPGFFNRKAAEQSAAEVALRELAK	LifeTein, USA

Supplemental table 4 | RNA substrates used for EMSA studies

Name	Sense (5'-3')	antisense(5'- 3')	Supplier
amiR28	TCGAATAAGATACTATACGTT	AACGTATACTATCTTATTCGA	IDT, USA
amiR72	TCGAACAGGGCGCCAGTGCCC	GGGCACTGCCGCCCTGTTCGA	IDT, USA
amiR52	TCGAACTAGGTCTCAGTGCTC	GAGCACTGTGACCTAGTTCGA	IDT, USA
amiRscr52	TTATTAATTTCGGCCCCGGCG	CGCCGGGCCCGAAATTAATAA	IDT, USA
miR528_stemloop _modified	AGUGGAAGAGGCAUGCAGAGGAGGAAACUCCUGUGCUUGCCUCUUCCAUU		IBA, Germany
